# Comparative Peptidomic Identification of a Noncanonical PSK3 Precursor‐Derived Peptide That Enhances Antiviral Defence Through MAPK‐Dependent ROS Signalling in Tobacco

**DOI:** 10.1111/mpp.70361

**Published:** 2026-11-11

**Authors:** Bin Yong, Shiming Lin, Xiaowei Liu, Peng Liu, Tianye Zhang, Kaili Zhong, Jiaqian Liu, Qianjun Tang, Xiangping Zhou, Hailin Cai, Tianbo Liu, Jian Yang

**Affiliations:** ^1^ State Key Laboratory for Quality and Safety of Agro‐Products, Key Laboratory of Biotechnology in Plant Protection of MARA, Zhejiang Key Laboratory of Green Plant Protection, Institute of Plant Virology Ningbo University Ningbo China; ^2^ College of Plant Protection Hunan Agricultural University Changsha China; ^3^ Yongzhou Tobacco Company of Hunan Province Yongzhou China; ^4^ Changsha Tobacco Company of Hunan Province Changsha China; ^5^ Tobacco Research Institute of Hunan Province Changsha China

**Keywords:** peptidomics, phytosulfokine peptide, plant antiviral defence, *Potyvirus*, ROS burst

## Abstract

Plant viral diseases cause substantial agricultural losses, yet the endogenous signals contributing to antiviral immunity remain poorly understood. In this study, we performed comparative peptidomic profiling of three tobacco cultivars with contrasting resistance to potato virus Y (PVY), namely, Coker176 with high resistance, CS01 with moderate resistance, and NC95 with high susceptibility, and identified distinct resistance‐associated peptide occurrence and accumulation patterns. Among the candidate peptides derived from known signalling‐peptide precursors, PSK #2 exhibited significant antiviral activity. Sequence analyses revealed that PSK #2 originates from a noncanonical region of the PSK3 precursor distinct from the conserved YIYTQ motif; therefore, it was designated a NbPSK3‐derived peptide (NbPSK3‐DP). Genetic analyses revealed that the NbPSK3 precursor positively regulates PVY resistance in tobacco, whereas its disruption increases shoot branching. Mechanistically, NbPSK3‐DP rapidly activates MPK3/MPK6 phosphorylation and induces a transient ROS burst. Functional analyses revealed that *NbSnRK2* and *NbACS1* contribute to NbPSK3‐mediated antiviral resistance. Phytohormone profiling revealed coordinated changes in jasmonic acid (JA)‐related metabolites, and exogenous JA treatment decreased PVY accumulation in *cr‐nbpsk3* plants, supporting the involvement of JA‐associated signalling. Additionally, both *NbPSK3* overexpression and exogenous NbPSK3‐DP application increased resistance to multiple tested plant viruses. Together, our findings identify a noncanonical bioactive peptide derived from the NbPSK3 precursor and demonstrate the utility of comparative peptidomics for identifying endogenous peptide regulators of antiviral immunity.

## Graphical abstract

This study identifies NbPSK3 as a PSK3‐derived non‐canonical peptide signal that enhances antiviral immunity in tobacco by activating MAPK signalling and ROS burst; shows potential for broader antiviral protection.

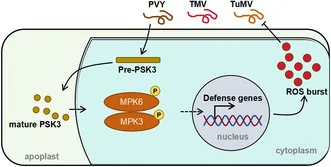

## 1 Introduction

Small peptides are among the smaller biologically active molecules in organisms, typically ranging from 2 to 100 amino acids in length, and function as key intercellular communicators that coordinate a wide range of developmental and physiological processes (Wheeler and Irving 2010; Matsubayashi 2014, 2018; Tavormina et al. 2015; Rzemieniewski and Stegmann 2022; Pastor‐Fernández et al. 2023; Xiao et al. 2025; Li et al. 2025). Small peptides are derived from larger precursor proteins through proteolytic processing, often accompanied by post‐translational modifications such as tyrosine sulphation, hydroxylation, proline hydroxylation, and glycosylation, which are essential for their bioactivity (Matsubayashi 2014; Tavormina et al. 2015; Zhang et al. 2024). Upon secretion into the apoplast, mature peptides are perceived by plasma membrane‐localized receptors, predominantly leucine‐rich repeat receptor‐like kinases (LRR‐RLKs), which transduce extracellular signals into intracellular responses (Matsubayashi 2018; Hou et al. 2021; Xiao et al. 2025). Among the diverse roles of plant peptides, their participation in immunity has attracted particular attention. Endogenous immune‐active peptides amplify defence signalling and elicit characteristic immune outputs, including reactive oxygen species (ROS) bursts, mitogen‐activated protein kinase (MAPK) activation, calcium influx, hormone crosstalk, and defence gene induction (Hou et al. 2021; Rzemieniewski and Stegmann 2022; Pastor‐Fernández et al. 2023).

Plants initiate innate immune responses through pattern‐triggered immunity (PTI), which is activated by the recognition of pathogen‐associated molecular patterns (PAMPs), and effector‐triggered immunity (ETI), with endogenous peptides serving as critical amplifiers and modulators of both pathways (Ngou et al. 2022; Hou et al. 2021; Yuan et al. 2021). Damage‐associated molecular patterns (DAMPs), many of which are peptide‐based, further integrate stress signals with immune activation (Yu et al. 2017; Wang, Yao, et al. 2020). DAMPs can be divided into constitutive DAMPs (cDAMPs), which perform primary cellular or structural functions and acquire signalling activity after damage‐dependent release or fragmentation, and inducible DAMPs (iDAMPs), including phytocytokines that primarily function as immune signals (Tanaka and Heil 2021). Representative cDAMPs include oligogalacturonides (OGs), extracellular ATP (eATP), extracellular NAD(P)^+^, and glutamate. These molecules activate local or systemic immune outputs, including Ca^2+^, ROS, MAPK activation, and defence gene expression (Benedetti et al. 2015; Wang et al. 2019; Harris and Mou 2024). Plant elicitor peptides (PEPs), which are perceived by plasma membrane‐localized PEP receptors (PEPRs), are among the best‐characterized immune peptides: They enhance innate immunity and promote both local and systemic defence responses in *Arabidopsis*, solanaceous plants, and wheat ( *Triticum aestivum* ) (Yamaguchi et al. 2006; Huffaker et al. 2006; Bartels et al. 2013; Ross et al. 2014; Klauser et al. 2015; Shen et al. 2017; Dong et al. 2025). Systemin, a peptide hormone produced in tomato in response to wounding and herbivory, activates the jasmonic acid (JA) pathway and systemic wound signalling (Ryan 2000). Collectively, endogenous peptides constitute a versatile layer of immune regulation. Wang, Yao, et al. (2020) employed transcriptomics‐ and proteomics‐based screening to identify 236 small secreted peptides (SSPs) in rice ( *Oryza sativa* ) in response to *Magnaporthe oryzae*, including rapid alkalinization factors (RALFs), phytosulfokines (PSKs), and an immune response peptide (IRP), that induce phenylalanine ammonia‐lyase (PAL) expression and activate MAPKs. Liu, Zhang, Liu, et al. (2025) demonstrated that the apoplastic signal peptide CLE7 increases antiviral resistance in *Nicotiana benthamiana* through a NbPXC3–NbMKK2–NbMPK4–NbEDT1 signalling cascade linked to abscisic acid (ABA) biosynthesis and that exogenous CLE7 application can suppress infection by multiple RNA viruses. A wound‐induced PR1‐derived peptide was also identified via peptidomics in tomato ( *Solanum lycopersicum* ), and subsequent studies demonstrated that XCP1 cleaves PR1 to release CAPE9, a peptide critical for systemic immunity in *Arabidopsis thaliana* (Chen et al. 2014, 2023). These findings collectively underscore that endogenous peptides in the apoplast occupy a strategic position integrating pathogen perception with downstream immune activation, and that omics, especially peptidomics, is a powerful discovery platform for identifying such regulators. Recently, integrated peptidogenomic, ribosome profiling, and high‐resolution mass spectrometry (MS) strategies have rapidly expanded the detectable plant peptidome and accelerated functional annotation across species (Zhang et al. 2025; Wang et al. 2026).

PSKs are a family of tyrosine‐sulphated peptide hormones that are widely distributed in higher plants. The canonical bioactive form, PSKα, has the sequence Tyr(SO₃H)‐Ile‐Tyr(SO₃H)‐Thr‐Gln (YIYTQ) and was first isolated from the conditioned medium of *Asparagus officinalis* mesophyll cell cultures as a potent cell proliferation‐promoting factor (Matsubayashi and Sakagami 1996). PSKα is processed from small secreted precursor polypeptides, typically 80–120 amino acids in length, that are conserved across diverse plant species and contain a highly conserved YIYTQ core motif (Lorbiecke and Sauter 2002; Yang et al. 2001; Yu et al. 2019; Wu et al. 2025). The biosynthesis of active PSKα signalling requires two sequential post‐translational events: tyrosine sulphation by tyrosylprotein sulfotransferase (TPST) in the Golgi apparatus and proteolytic cleavage by subtilisin‐like serine proteases (SBTs) in the apoplast (Matsubayashi et al. 1996; Komori et al. 2009; Stührwohldt et al. 2021; Wu et al. 2025). Tyrosine sulphation is critical for the high biological activity of canonical PSKα, as the removal of either sulphate group substantially decreases its activity, and the unsulphated peptide exhibits little or no activity in classical PSK bioassays (Matsubayashi and Sakagami 1996; Ladwig et al. 2015). In *Arabidopsis*, PSK is primarily perceived by PSKR1, with its paralogue PSKR2 playing a secondary role, while CYCLIC NUCLEOTIDE‐GATED CHANNEL 17 (CNGC17) and H^+^‐ATPases are also recruited to the receptor complex (Matsubayashi et al. 2006; Amano et al. 2007; Ladwig et al. 2015). Beyond its essential role in cell proliferation (Matsubayashi and Sakagami 1996), PSK has been shown to regulate a remarkably diverse array of developmental and physiological processes, including cell expansion, hypocotyl elongation (Stührwohldt et al. 2011), root growth (Kutschmar et al. 2009), pollen germination and pollen tube guidance (Stührwohldt et al. 2015), nodulation in legumes (Wang et al. 2015), fruit ripening (Fang et al. 2024), and biotic stress responses (Stührwohldt et al. 2021; Nagar et al. 2022).

The role of PSK peptides in plant immunity varies significantly depending on the type of pathogen, the host species, and the signalling partners involved. In *Arabidopsis*, PSK–PSKR1 attenuates PTI by suppressing salicylic acid (SA)‐mediated defences, leading to increased susceptibility to the biotrophic bacterial pathogen *Pseudomonas syringae* pv. *tomato*, but paradoxically increased resistance to the necrotrophic fungus *Alternaria brassicicola* (Igarashi et al. 2012; Mosher et al. 2013). PSK–PSKR1 signalling, which is also activated by intracellular Ca^2+^ release, initiates an auxin‐dependent cascade that increases resistance to *Botrytis cinerea* in tomato (Zhang et al. 2018), while the E3 ubiquitin ligase PUB12 fine‐tunes this immune response by targeting PSKR1 for ubiquitination and degradation (Hu et al. 2023). Moreover, PSK mediates resistance to both *M. oryzae* and bacterial blight, with overexpression promoting PR gene expression and ROS accumulation without growth penalties (Liu, Zhang, Zeng, et al. 2025). Similar findings have also been reported regarding Verticillium wilt resistance in cotton ( *Gossypium hirsutum* ) (Ogawa‐Ohnishi et al. 2022). Additionally, PSK signalling through the PSKR1–BAK1 coreceptor complex fully protects *Arabidopsis* from fumonisin B1 (FB1)‐induces cell death, and the bacterial PAMP flg22 suppresses FB1 toxicity by activating PSK signalling downstream of FLS2 receptor activation, revealing a mechanistic link between PAMP perception and the suppression of PSK‐mediated cell death (Alqarni et al. 2026). Betulinic acid activates the PSK–PSKR signalling pathway through upregulation of *NbPSK3* expression under turnip mosaic virus (TuMV) infection in *Nicotiana benthamiana*, establishing a direct link between PSK signalling and antiviral defence (Xiang et al. 2025). Together, these cross‐species comparisons reveal that while the role of PSK in immunity is conserved, the specific hormonal and molecular interactions through which it operates are adapted to the pathosystem context.

The biological information encoded by a peptide precursor is not necessarily restricted to a single canonical mature peptide. Several plant peptide systems provide precedents for the presence of multiple or previously unrecognized bioactive sequences within a single precursor or larger protein. For example, a single hydroxyproline‐rich systemin precursor can give rise to multiple defence‐signalling peptides (Pearce et al. 2001), whereas the soybean subtilisin‐like protein harbouring GmSubPep contains an embedded 12‐amino‐acid cryptic peptide that can activate defence‐related genes (Pearce et al. 2010). Quantitative peptidomics led to the identification of CAPE1, an 11‐amino‐acid immune‐regulatory peptide derived from the C‐terminal region of tomato PR‐1, illustrating how endogenous peptidomic analyses can reveal bioactive peptides that are not evident from conventional protein annotation (Chen et al. 2014). Similarly, regions of tomato Prosystemin located outside the canonical C‐terminal Systemin sequence retain biological activity and can induce defence‐related responses when experimentally tested (Molisso et al. 2022). Together, these findings indicate that precursor proteins can harbour biologically active regions other than their best‐characterized mature peptide products. In contrast, the biological activity of PSK precursors has thus far been attributed predominantly to the conserved sulphated PSK motif. Whether regions outside the canonical YIYTQ motif of PSK precursors can generate additional bioactive peptide signals remains largely unexplored.

Viral diseases in plants are among the most important biotic constraints on global agricultural productivity, causing substantial annual yield losses across staple food crops, vegetables, and economically important industrial crops (Jones and Naidu 2019; Scholthof et al. 2011). Unlike fungal or bacterial pathogens, plant viruses exploit host cellular machinery as obligate intracellular parasites and are poorly controlled by conventional chemical pesticides, making the prevention and management of viral diseases a persistent challenge in modern agriculture (Tatineni and Hein 2023). Major crop viruses, including members of the *Potyviridae*, *Tobamoviridae*, *Begomoviridae*, *Cucumoviridae*, and *Luteoviridae* families, collectively threaten the production of economically important crops worldwide (Scholthof et al. 2011; Tatineni and Hein 2023). The rapid evolution of plant viruses through mutation, recombination, and reassortment further complicates resistance management, allowing viruses to overcome host resistance genes and rendering conventional breeding‐based approaches unstable over time (Quenouille et al. 2013). Current disease management strategies—including host resistance breeding, vector control through insecticides, and cultural practices—remain insufficient to provide durable broad‐spectrum protection, and the development of novel antiviral strategies with strong mechanistic foundations is urgently needed (Jones and Naidu 2019).

Therefore, the identification of endogenous peptides that regulate antiviral resistance, together with the elucidation of their underlying mechanisms, is particularly important for the development of environmentally friendly approaches for viral disease management. In this study, the comparative peptidomic profiling of tobacco ( *Nicotiana tabacum* ) accessions with contrasting resistance to potato virus Y (PVY) revealed a resistance‐associated peptide derived from a noncanonical region of the NbPSK3 precursor, here designated NbPSK3‐derived peptide (NbPSK3‐DP). Functional analyses revealed that NbPSK3‐DP increases resistance to PVY and multiple tested plant viruses. NbPSK3‐associated signalling activated MAPK phosphorylation and induced a rapid ROS burst. In addition, *NbSnRK2* and *NbACS1* contributed to NbPSK3‐mediated antiviral resistance, while phytohormone profiling and exogenous JA treatment further supported the involvement of JA‐associated signalling. Overall, our findings underscore the value of comparative peptidomics in identifying endogenous resistance‐associated peptides and provide a theoretical foundation for the environmental management of plant viral diseases.

## 2 Results

### 2.1 Global Peptidomic Profiling of Tobacco in Response to PVY Infection

To systematically capture the endogenous peptide dynamics associated with antiviral resistance, we performed high‐resolution LC–MS/MS‐based peptidomics. To identify tobacco varieties suitable for small‐peptide omics analysis, we screened 60 tobacco germplasm accessions for PVY resistance (Figure S1). Among these 60 accessions, 3 accessions were highly resistant, 10 were moderately resistant, 23 were moderately susceptible, and 24 were highly susceptible. To identify peptides associated with PVY resistance in tobacco, we selected three germplasm lines—Coker176, which is highly resistant; CS01, which is moderately resistant; and NC95, which is highly susceptible—for peptidomic profiling and analysis following PVY infection. The biological replicates were highly reproducible within the cultivars, as shown by strong within‐group correlations (Figure 1A). Principal component analysis separated the samples according to their resistance phenotypes. In addition to PC1 (49.7%), Coker176 and CS01 were clearly distinguished from NC95, indicating that they shared resistance‐associated molecular features (Figure 1B). Along PC2 (34.1%), Coker176 was further separated from CS01 and NC95, suggesting that it may harbour additional resistance‐specific regulatory components (Figure 1B). Across all the samples, 31,260 peptides were identified, most of which were 10–20 amino acids in length and 1000–2000 Da in molecular weight; 30,855 of these peptides were traced to 6635 prepropeptides, including 1804 peptides derived from 457 prepropeptides of unknown function, while 9 peptides from 3 prepropeptides belonged to the known peptide families RALF, PSK, and GASA (Figure 1C; Figure S2). Given the importance of secreted peptides in plant immunity, we evaluated peptides with secreted peptide features in our dataset, defined as those derived from prepropeptides carrying a signal peptide and shorter than 300 amino acids, and found that only 4.5% met these criteria (Figure 1D). These results suggest that PVY resistance in tobacco is associated with distinct peptide accumulation patterns and that beyond the limited number of canonical secreted peptides, additional uncharacterized peptides may also contribute to antiviral responses.

**FIGURE 1:**
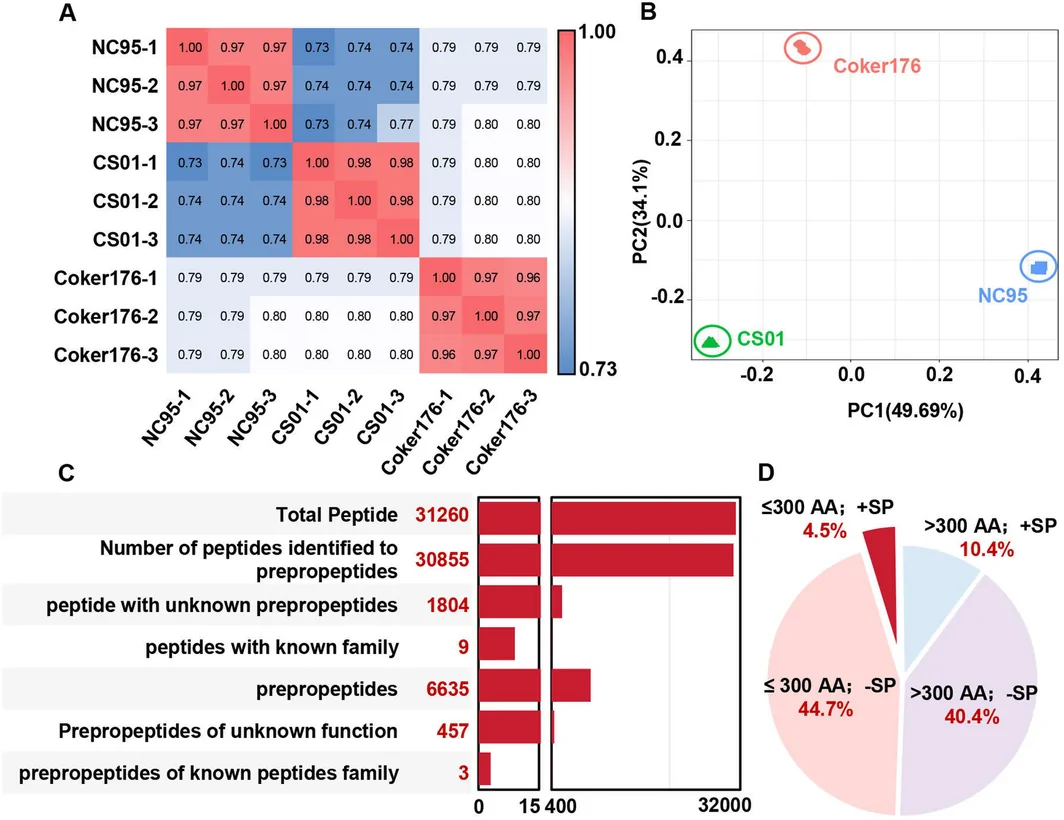
Global peptidome profiling of PVY‐resistant and PVY‐susceptible cultivars. (A) Pearson correlation heatmap of peptide abundance profiles from three biological replicates of each PVY‐infected *Nicotiana tabacum* cultivar: Coker176, CS01 and NC95. (B) Principal component analysis of peptide abundance profiles. The percentages of variance explained by the first two principal components are indicated on the axes. (C) Summary of peptide identification and mapping to precursor proteins (preproproteins). (D) Classification of peptide precursor proteins by length (≤ 300 amino acids or > 300 amino acids) and presence/absence of predicted signal peptides (+SP or −SP). Three independent biological replicates were analysed for each cultivar.

### 2.2 Identification of Candidate Resistance‐Associated Peptides in Tobacco

Following the global characterization of the peptidome in the three tobacco accessions, we next compared the peptide distribution among all the accessions to identify peptides potentially associated with PVY resistance. Venn analysis revealed that 9523 peptides (30.5%) were shared among Coker176, CS01, and NC95, whereas 3659 (11.7%), 5044 (16.1%), and 5818 (18.6%) peptides were uniquely detected in Coker176, CS01, and NC95, respectively (Figure 2A). In addition, 2892 peptides (9.3%) were shared only between the two resistant lines, Coker176 and CS01, while 2627 (8.4%) and 1697 (5.4%) were shared only between Coker176 and NC95 and between CS01 and NC95, respectively (Figure 2A). The classification of peptide occurrence and accumulation patterns revealed that 11,595 peptides were specific to the resistant group, whereas 5818 peptides were detected exclusively in the susceptible cultivar (Figure 2B). Furthermore, 3199 and 5261 peptides were enriched in the resistant and susceptible groups, respectively, showing at least 1.5‐fold greater accumulation than the opposite group (Figure 2B). Further classification by prepropeptide features revealed that only a limited fraction of these peptides were derived from putative prepropeptides of secreted peptide (+SP and ≤ 300 amino acids), with 53–100 peptides across the different groups, whereas the majority originated from precursors lacking canonical secreted peptide features (Figure S3). Functional enrichment analysis of the corresponding precursor proteins revealed distinct biological signatures among the resistance‐ and susceptibility‐associated secreted peptide groups (Figure 2C). S‐specific and S‐enriched precursors were associated mainly with defence responses, calcium‐mediated signalling, and oxidative stress, whereas R‐specific precursors were more strongly related to cell wall organization and metabolic processes. Notably, R‐enriched precursors were associated with defence response, response to wounding, phenylpropanoid biosynthesis, plasmodesma‐mediated intercellular transport, and cell wall‐related processes (Figure 2C). Given that previous studies have shown that known peptide families, such as RALF and PSK, can participate in plant immune activation, we examined the accumulation patterns of peptides assigned to known families. These peptides showed clear cultivar‐specific accumulation patterns across Coker176, CS01, and NC95, and notably, only RALF #5, PSK #1, and PSK #2 were detected in the resistant varieties but not in the susceptible variety (Figure 2D). Therefore, RALF #5, PSK #1, and PSK #2 were selected as candidate resistance‐associated peptides for further investigation.

**FIGURE 2:**
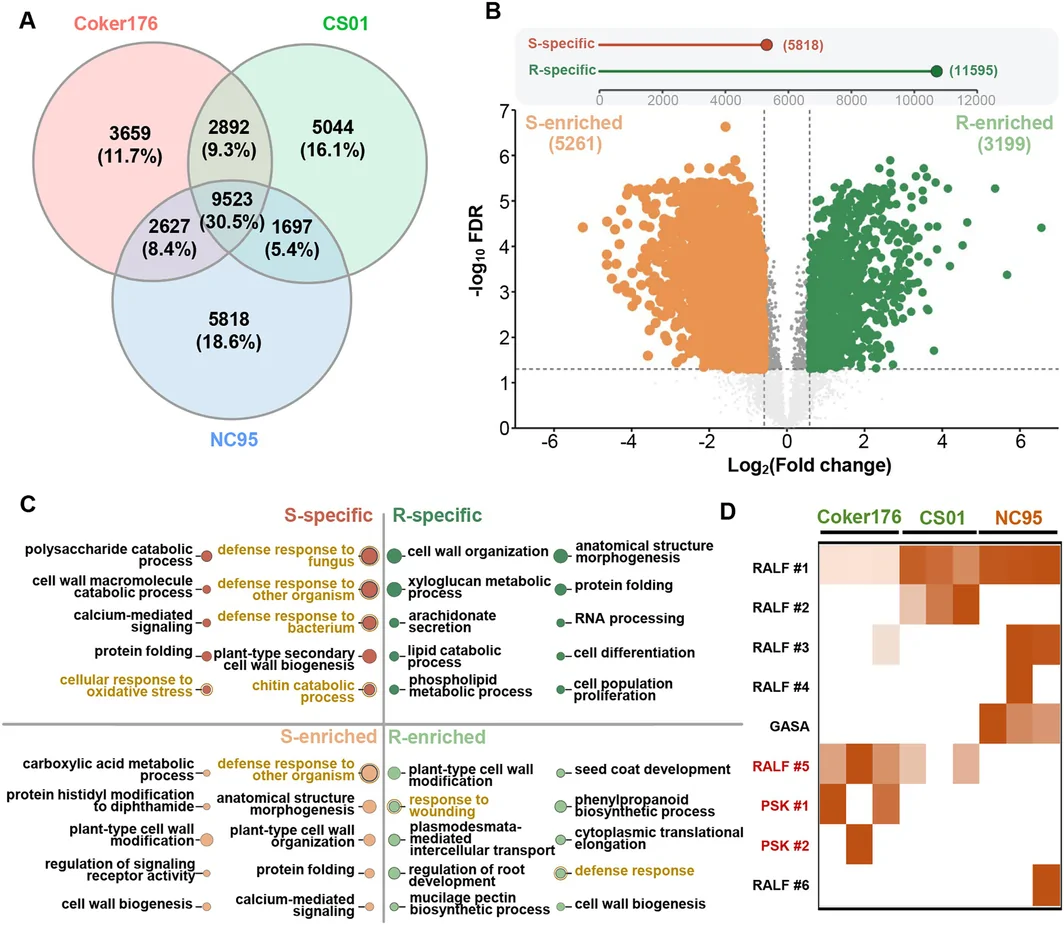
Identification of resistance‐associated endogenous peptide candidates in tobacco. (A) Venn diagram showing shared and cultivar‐specific peptides in Coker176, CS01, and NC95. (B) Classification of resistance‐associated peptide candidates according to their occurrence and differential accumulation between resistant (Coker176 and CS01) and susceptible (NC95) cultivars. Peptides detected specifically in resistant or susceptible materials were classified as R‐specific or S‐specific, respectively, whereas peptides showing ≥ 1.5‐fold differences in abundance were classified as R‐enriched or S‐enriched. (C) Functional enrichment analysis of precursor proteins corresponding to R‐specific, R‐enriched, S‐specific, and S‐enriched candidate peptides. Representative biological process and molecular function terms are shown. These precursors have a predicted signal peptide and are ≤ 300 amino acids long. (D) Abundance profiles of peptides assigned to the PSK, RALF and GASA families. The candidates selected for subsequent assays, RALF #5, PSK #1 and PSK #2, are highlighted in red. Each cultivar is represented by three biological replicates.

### 2.3 NbPSK3‐Derived Peptide Is a Noncanonical Precursor‐Derived Peptide Secreted Into the Apoplast

To determine whether RALF #5, PSK #1, and PSK #2 possess antiviral activity against PVY, we chemically synthesized these peptides and evaluated their effects in biological assays. At 5 days post‐inoculation (dpi), compared with those in the water control, PVY‐GFP accumulation and relative expression of PVY *CP* were markedly reduced in the tissues treated with PSK #2, whereas the inhibitory effects of PSK #1 and RALF #5 did not show a comparable inhibitory effect (Figure 3A,B; Figure S4). On the basis of these results, PSK #2 was selected as the resistance‐associated candidate peptide from tobacco resistant lines for further investigation. Phylogenetic analysis of sequences from *Arabidopsis* and *N. benthamiana* revealed that the precursor protein containing PSK #2 clustered within the AtPSK3/PSK3‐like clade and was designated NbPSK3. Because PSK #2 originates from a noncanonical region of the NbPSK3 precursor outside the conserved YIYTQ motif, it was renamed NbPSK3‐derived peptide (NbPSK3‐DP) (Figure 3C). We next examined the subcellular localization of NbPSK3 to determine whether it shows the typical trafficking pattern of a secreted peptide precursor. NbPSK3‐RFP, the signal peptide‐deleted form of NbPSK3^ΔSP^‐RFP, and the apoplast marker AtPIP1‐RFP were transiently expressed in *N. benthamiana* leaves together with the plasma membrane marker NbCERK. Under normal conditions, the location of both AtPIP1 and NbPSK3 substantially overlapped with NbCERK, which is consistent with their association with the cell periphery (Figure 3D). However, after plasmolysis, the fluorescence signals of AtPIP1 and NbPSK3 clearly separated from those of NbCERK and were detected in the apoplastic space, whereas NbPSK3^ΔSP^ largely remained associated with the retracted plasma membrane (Figure 3D). Additionally, the accumulation of NbPSK3‐FLAG was detected in the apoplastic fluids of the assayed *N. benthamiana* leaves (Figure 3E). Together, these results indicate that PSK #2 is a PSK3‐like peptide derived from the precursor NbPSK3 and that NbPSK3 is secreted into the apoplast in a signal peptide‐dependent manner.

**FIGURE 3:**
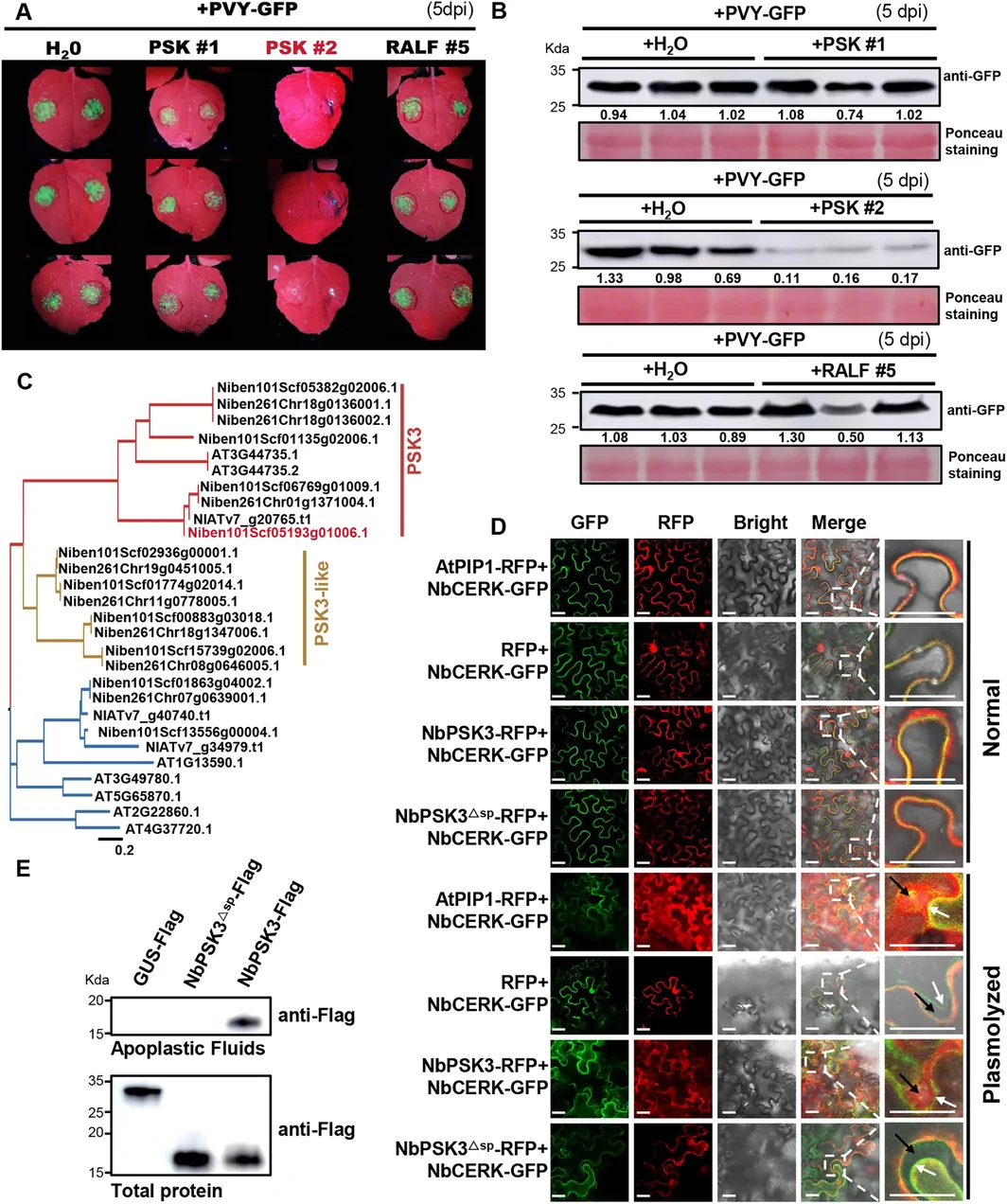
Antiviral activity of NbPSK3‐derived peptide (NbPSK3‐DP) and apoplastic localization of the NbPSK3 precursor. (A) PVY‐GFP fluorescence in *Nicotiana benthamiana* leaves treated with the synthetic candidate peptides PSK #1, PSK #2 or RALF #5. Water treatment was used as the control. Images were recorded at 5 days post‐inoculation (dpi). (B) Western blot analysis of PVY infection in the inoculated leaves of *N. benthamiana* treated with candidate peptides at 5 dpi using GFP antibody. Ponceau S staining shows protein loading. Values beneath the bands indicate relative band intensities normalized to the corresponding loading control. (C) Phylogenetic analysis of PSK homologues in *Arabidopsis* and *Nicotiana benthamiana*. The precursor containing PSK #2 clustered within the AtPSK3/PSK3‐like clade and was designated NbPSK3 (highlighted in red font). PSK #2 originates from a distinct region outside the canonical YIYTQ motif and was therefore renamed NbPSK3‐derived peptide (NbPSK3‐DP). (D) Subcellular localization of NbPSK3 in *N. benthamiana* leaves under normal and plasmolysed conditions. NbPSK3 without signal peptide (NbPSK3^ΔSP^). The black arrows indicate apoplastic spaces, and white arrows indicate the plasma membrane. Bar, 10 μm. Each experiment was repeated three times independently with similar results. (E) Anti‐FLAG immunoblot analysis of apoplastic fluid and total‐protein fractions from leaves expressing NbPSK3 and NbPSK3^ΔSP^.

### 2.4 NbPSK3 Increases PVY Resistance While Repressing Shoot Branching in Tobacco

To investigate the role of NbPSK3 in tobacco resistance to PVY infection, an *NbPSK3*‐overexpressing line (OE*‐NbPSK3*) and a CRISPR/Cas9‐edited mutant line (*cr‐nbpsk3*) were generated. Sequencing analysis of the target region in the edited plants revealed insertion/deletion (indel) mutations around the sgRNA target sites, resulting in frameshifts and premature termination codons, which could prevent translation of the full‐length NbPSK3 preproprotein (Figure S5), confirming the successful generation of *NbPSK3* knockout lines. Under standard growth conditions, compared with the wild‐type plants, the OE*‐NbPSK3* plants exhibited no overt developmental abnormalities (Figure 4A). Conversely, the *cr‐nbpsk3* plants presented a bushier phenotype with increased lateral branching (Figure 4B). Quantitative analysis revealed no significant difference in plant height among wild‐type, OE*‐NbPSK3*, and *cr‐nbpsk3* plants (Figure 4C). The number of branches was significantly increased in the *cr‐nbpsk3* plants compared with the wild‐type, whereas the number of branches did not significantly differ between the OE*‐NbPSK3* plants and the wild‐type plants (Figure 4D). No significant difference in thousand‐seed weight was detected among the three genotypes (Figure 4E). The results obtained demonstrate that disruption of NbPSK3 has a predominant effect on shoot branching under normal growth conditions.

**FIGURE 4:**
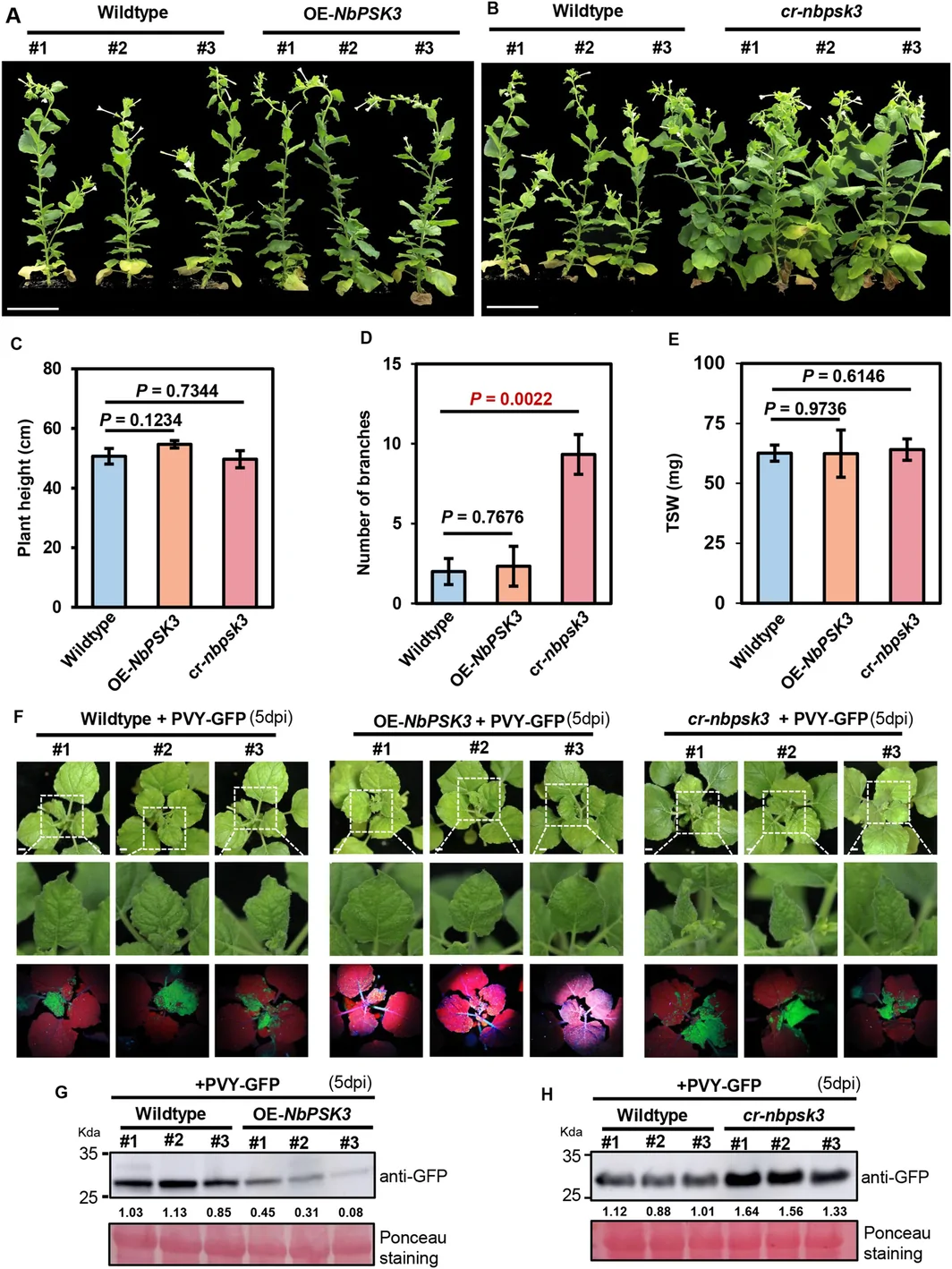
NbPSK3 negatively regulates shoot branching while enhancing PVY resistance. (A) Representative plant phenotypes of wild type and *NbPSK3* overexpression (OE*‐NbPSK3*) lines. (B) Representative plant phenotypes of wild type and *NbPSK3*‐edited mutants (*cr‐nbpsk3*). In (A) and (B), bar = 10 cm. (C) The plant height of wild type, OE*‐NbPSK3*, and *cr‐nbpsk3* lines. (D) The number of branches of wild type, OE*‐NbPSK3*, and *cr‐nbpsk3* lines. (E) The thousand‐seeds weight (TSW) of wild type, OE*‐NbPSK3*, and *cr‐nbpsk3* lines. In (C)–(E), statistical significance was assessed using *t*‐tests; each bar corresponds to the mean value ± SD. (F) Systemic symptoms and GFP fluorescence in systemic leaves of wild type, OE*‐NbPSK3*, and *cr‐nbpsk3* mutants at 5 days post‐inoculation (dpi) under PVY infection. Bar, 1 cm. (G) Western blot analysis of PVY accumulation in the systemic leaves of wild type and OE*‐NbPSK3* using GFP antibody at 5 dpi. (H) Western blot analysis of PVY accumulation in the systemic leaves of wild type and *cr‐nbpsk3* using GFP antibody at 5 dpi. In (G) and (H), Ponceau S staining is used to show sample loadings; values beneath the bands indicate relative band intensities normalized to the corresponding loading control.

Following inoculation with PVY‐GFP, compared with wild‐type plants, OE*‐NbPSK3* plants presented fewer infection symptoms and weaker GFP fluorescence signals at 5 dpi. In contrast, the *cr‐nbpsk3* plants presented stronger GFP fluorescence (Figure 4F). Compared with those in wild‐type plants, the PVY accumulation and relative expression of PVY *CP* were lower in OE*‐NbPSK3* plants but higher in *cr‐nbpsk3* plants (Figure 4G,H; Figure S6A,B). The overexpression of *NbPSK3* therefore led to a decrease in PVY accumulation, whereas the loss of *NbPSK3* resulted in an increase in PVY. In conclusion, the results demonstrate that NbPSK3 positively regulates the *N. benthamiana* response to PVY infection and participates in the regulation of shoot branching.

### 2.5 Transcriptomic and Functional Analyses Identify SnRK2 and ACS1 as Components of NbPSK3‐Mediated Antiviral Defence

To explore the molecular basis of NbPSK3‐mediated antiviral resistance, we first performed a transcriptome analysis of OE*‐NbPSK3* and wild‐type plants during PVY infection. The results demonstrated high reproducibility among biological replicates, with correlation coefficients ranging from 0.853 to 0.907 (Figure S7A). Principal component analysis (PCA) clearly distinguished OE*‐NbPSK3* from wild‐type samples, indicating the presence of distinct transcriptional profiles between the two genotypes during PVY infection (Figure S7B). A total of 506 differentially expressed genes (DEGs) were identified, including 154 that were overexpressed and 352 that were expressed at lower levels in the OE*‐NbPSK3* plants than in the wild‐type plants (Figure S7C,D). A subsequent functional enrichment analysis of these DEGs revealed significant enrichment in defence‐ and hormone‐related biological processes, including the defence response, immune response, innate immune response, and cellular response to ROS (Figure 5A). KEGG analysis demonstrated that these genes were enriched in several defence‐associated pathways, including the MAPK signalling pathway in plants, plant hormone signal transduction, and plant–pathogen interactions (Figure 5B). Notably, among the DEGs associated with the MAPK signalling pathway in plants, only *NbSnRK2* and *NbACS1* were upregulated (Table S1). Reverse transcription‐quantitative PCR (RT‐qPCR) analysis confirmed that compared with the wild type, the plants overexpressing *NbPSK3* also had significantly increased expression of *NbSnRK2* and *NbACS1* (Figure 5C). To determine whether these genes contribute to NbPSK3‐mediated antiviral resistance, *NbSnRK2* or *NbACS1* was silenced in OE‐*NbPSK3* plants using a tobacco rattle virus (TRV)‐based virus‐induced gene silencing (VIGS) system (Figure S8). Compared with the OE*‐NbPSK3* control plants, the silencing of either gene resulted in markedly increased PVY‐GFP fluorescence (Figure 5D). Consistent with the fluorescence phenotype, immunoblot analysis revealed increased accumulation of PVY‐GFP in *NbSnRK2*‐ or *NbACS1*‐silenced OE‐*NbPSK3* plants compared with OE‐*NbPSK3* control plants (Figure 5E). Collectively, these results indicate that *NbSnRK2* and *NbACS1* contribute to NbPSK3‐mediated resistance to PVY and are involved in NbPSK3‐associated defence signalling.

**FIGURE 5:**
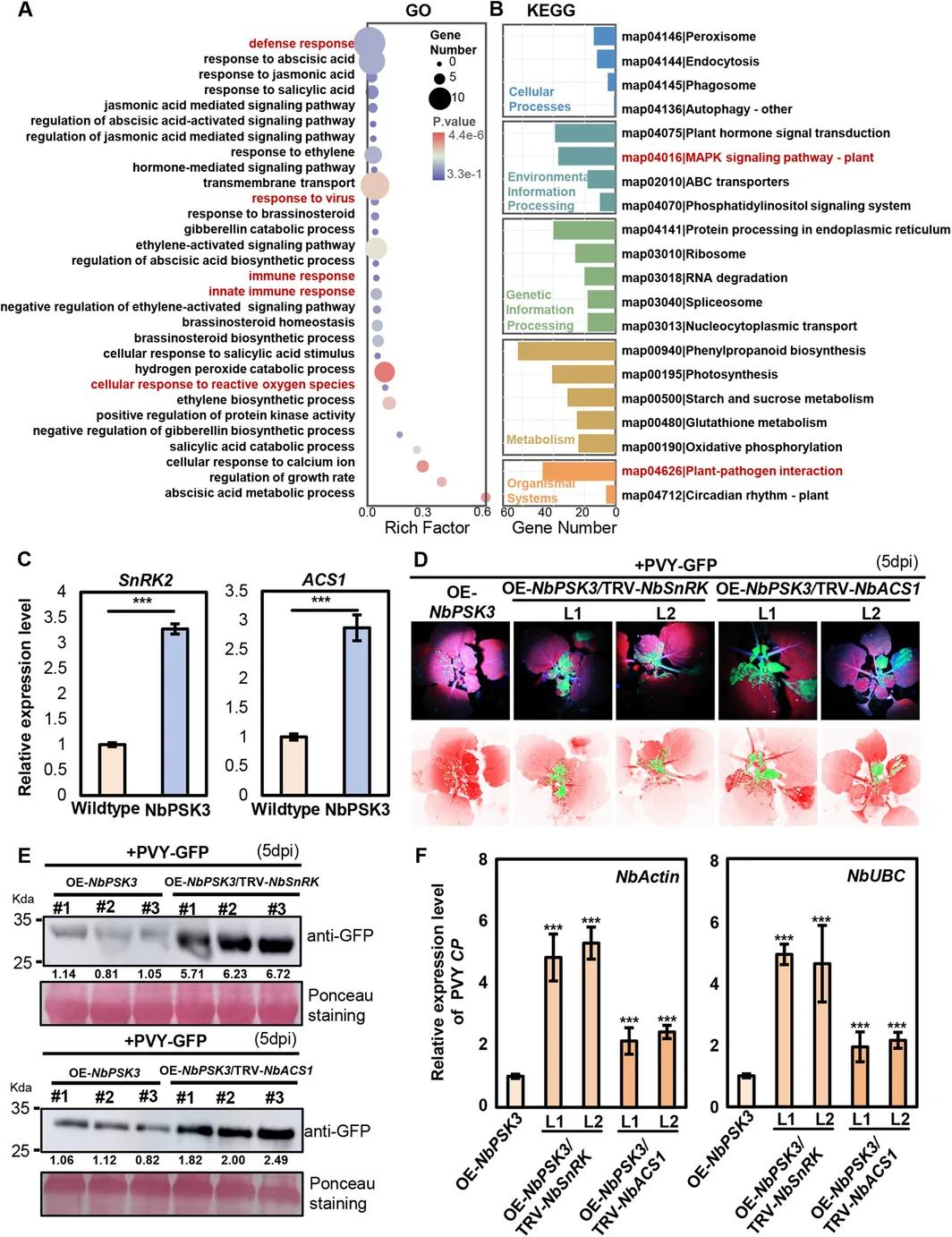
Transcriptomic and functional analyses identify NbSnRK2 and NbACS1 as components of NbPSK3‐mediated antiviral defence. (A) Gene Ontology (GO) enrichment analysis of differentially expressed genes (DEGs) in OE*‐PSK3* plants following PVY infection at 5 days post‐inoculation (dpi). (B) KEGG pathway enrichment of DEGs in OE*‐PSK3* plants following PVY infection at 5 dpi. In (A) and (B), plant immune‐related pathways are highlighted in red font. (C) Relative expression level of *NbSnRK2* and *NbACS1* in *Nicotiana benthamiana* leaves of wild‐type and OE*‐PSK3* following PVY infection at 5 dpi. Total RNA from wild‐type and OE*‐PSK3* plants was subjected to reverse transcription‐quantitative PCR (RT‐qPCR). (D) PVY‐GFP fluorescence in OE‐*NbPSK3* plants following TRV‐mediated silencing of *NbSnRK2* or *NbACS1*. OE‐*NbPSK3* plants carrying the corresponding TRV control were used as controls. (E) Immunoblot analysis of PVY‐GFP accumulation in *NbSnRK2*‐ and *NbACS1*‐silenced OE‐*NbPSK3* plants. (F) RT‐qPCR analysis of PVY *CP* accumulation in the indicated plants. In (C) and (F), “***” indicates significant difference (*p* < 0.01) in one‐way ANOVA. Each bar corresponds to the mean value ± SD of three biological replicates. PVY *CP* accumulation was independently normalized to *NbActin* (left) and *NbUBC* (right), shown in the left and right bar plots, respectively.

### 2.6 NbPSK3‐DP Induces ROS Burst Through the Activation of MAPK Signalling

The potential of NbPSK3 to activate the MAPK signalling pathway was determined through the extraction of total proteins at various time points after treatment and subsequent analysis using an anti‐pTEpY antibody. Compared with water treatment, NbPSK3‐DP treatment resulted in increased pMPK6 and pMPK3 phosphorylation levels, with the strongest pMPK6 signal detected at 15 min, and sustained activation was still observable at 30 and 60 min (Figure 6A). To further investigate whether PSK activates the host immune response via MAPK signalling mediated by MPK3 and MPK6, we silenced *MPK3* and *MPK6* in OE*‐NbPSK3* plants. Viral accumulation increased significantly following the silencing of *MPK3* and *MPK6* (Figure 6B–D). Similarly, NbPSK3 treatment did not decrease viral accumulation in *mpk3* ^*−/−*^ or *mpk3* ^*−/−*^ *mpk6‐RNAi* mutants (Figure 6B–D). To determine whether NbPSK3‐DP could stimulate ROS production, leaf discs were treated with NbPSK3‐DP, flg22, or water. NbPSK3‐DP rapidly induced a ROS burst, which peaked at approximately 20–25 min after treatment, whereas the level of ROS in the water control remained low throughout the detection period (Figure 6E). Quantification of total ROS production further revealed that compared with the water control treatment, the NbPSK3‐DP treatment resulted in significantly greater ROS accumulation, although the response was weaker than that induced by flg22 (Figure 6F). Consistent with these results, 3,3′‐diaminobenzidine (DAB) staining revealed pronounced ROS accumulation in the NbPSK3‐DP‐treated leaves at 4, 6, and 8 h after treatment, with the strongest staining observed at approximately 6 h, whereas only weak staining was observed in the water‐treated controls (Figure 6G). To further determine whether the NbPSK3‐DP‐induced increase in ROS depends on MAPK signalling, DAB staining was performed on wild‐type, *mpk3*, and *mpk3^−/−^mpk6‐*RNAi plants following PVY infection, and the results revealed that no ROS accumulation was observed in either *mpk3* or *mpk3^−/−^mpk6‐RNAi* plants with or without NbPSK3‐DP treatment (Figure 6H). In summary, the results suggest that NbPSK3‐DP induces a ROS burst by activating the MAPK signalling pathway.

**FIGURE 6:**
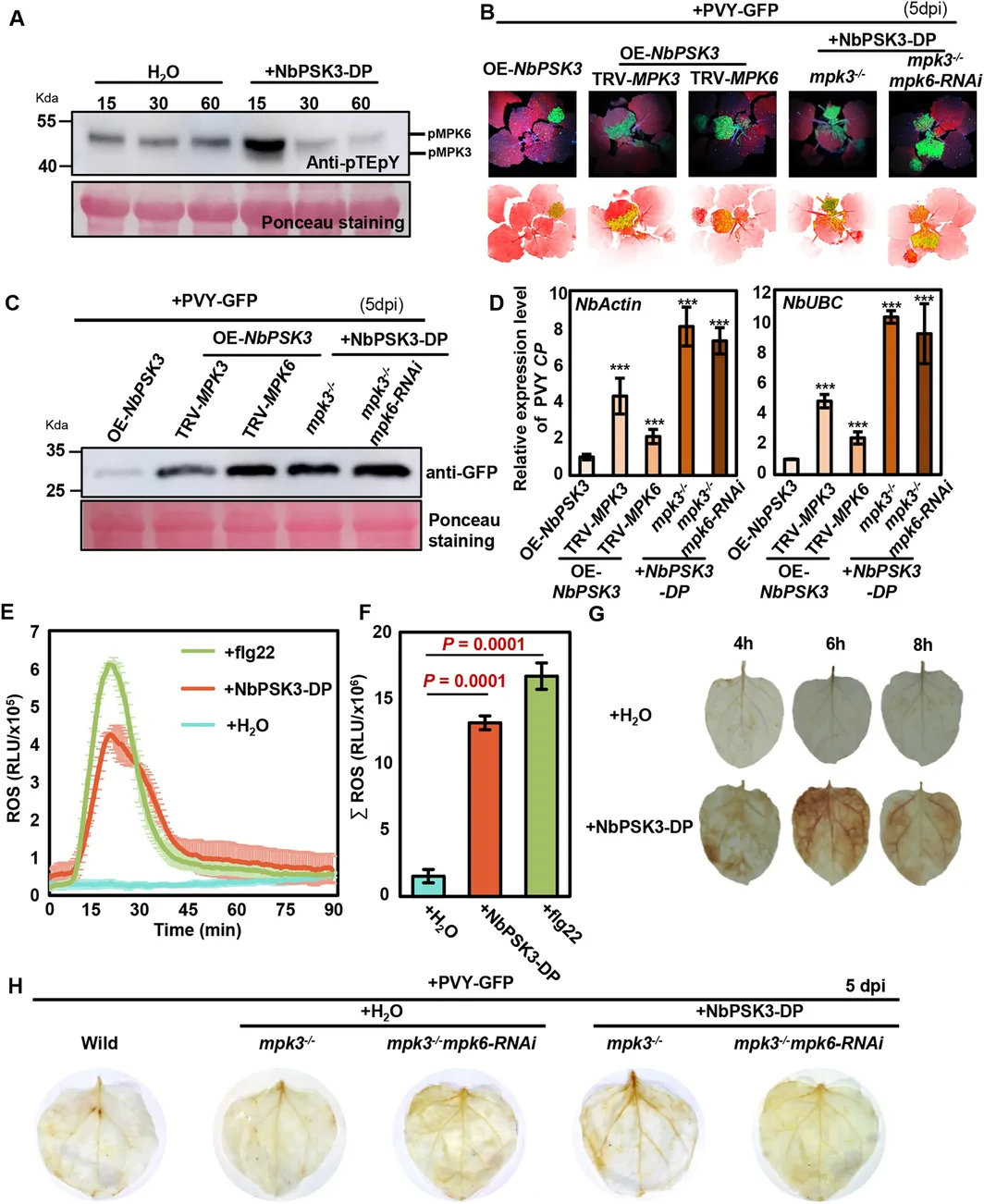
NbPSK3‐DP activates MAPK signalling and induces a rapid reactive oxygen species (ROS) response. (A) Immunoblot analysis of MPK3/MPK6 phosphorylation in *Nicotiana benthamiana* leaves at 15, 30 and 60 min after treatment with NbPSK3‐DP or water, using an anti‐pTEpY antibody. The bands assigned to phosphorylated MPK3 and MPK6 are indicated. Ponceau S staining shows protein loading. (B) Representative plant phenotypes of OE*‐NbPSK3*, TRV‐*MPK3*/TRV‐*MPK6* in OE*‐NbPSK3*, *mpk3* ^*−/−*^, and *mpk3* ^*−/−*^ *mpk6*‐RNAi mutants under PVY‐GFP infection at 5 days post‐inoculation (dpi). (C) PVY‐GFP accumulation in OE*‐NbPSK3*, TRV‐*MPK3*/TRV‐*MPK6* in OE*‐NbPSK3*, *mpk3* ^*−/−*^, and *mpk3* ^*−/−*^ *mpk6*‐RNAi mutants at 5 dpi by western blot. (D) Reverse transcription‐quantitative PCR analysis of PVY *CP* accumulation in OE*‐NbPSK3*, TRV‐*MPK3*/TRV‐*MPK6* in OE*‐NbPSK3*, *mpk3* ^*−/−*^, and *mpk3* ^*−/−*^ *mpk6*‐RNAi mutants at 5 dpi. PVY *CP* accumulation was independently normalized to *NbActin* and *NbUBC*, shown in the left and right bar plots, respectively. “***” indicate significant difference (*p* < 0.01) in one‐way ANOVA. Each bar corresponds to the mean value ± SD of three biological replicates. (E) Kinetics of ROS production in *N. benthamiana* leaf discs following treatment with NbPSK3‐DP, flg22, or water. Chemiluminescence was recorded at 30‐s intervals. (F) Cumulative ROS‐associated chemiluminescence over the recording period in (E). *p*‐values from *t*‐tests are shown for the indicated comparisons. Three biological replicates were used for the ROS measurements. (G) ROS accumulation was detected in NbPSK3‐treated *N. benthamiana* leaves at different time points (4, 6 and 8 h) using 3,3′‐diaminobenzidine (DAB) staining. Water treatment served as the control. (H) DAB staining of PVY‐GFP‐infected wild‐type, *mpk3* ^*−/−*^, and *mpk3* ^*−/−−*^ *mpk6*‐RNAi plants at 5 dpi, with the water and NbPSK3‐DP treatments indicated.

### 2.7 Exogenous JA Restores Antiviral Resistance in *cr‐nbpsk3* Plants

Given the close association between MAPK signalling and phytohormone regulation in plant immunity, we compared the phytohormone profiles of wild‐type and OE‐*NbPSK3* plants following PVY‐GFP infection. Distinct changes were observed in the expression of several hormone classes, including jasmonates, cytokinins, gibberellins, auxin, ABA, SA, and the ethylene precursor ACC (Figure 7A). Among these metabolites, JA‐related metabolites exhibited a particularly coordinated pattern in OE‐*NbPSK3* plants: the levels of JA and its bioactive conjugate JA‐Ile consistently increased, whereas those of JA precursor OPDA tended to decrease. In contrast, changes in other hormone classes were mainly restricted to individual metabolites or showed less coordinated patterns across the corresponding hormone group. Given the established role of JA signalling in plant defence and the coordinated alteration of multiple JA‐related metabolites observed here, JA was selected for subsequent functional validation. To determine whether JA contributes to NbPSK3‐mediated antiviral resistance, *cr‐nbpsk3* plants were treated with exogenous JA prior to PVY‐GFP infection. JA treatment markedly reduced GFP fluorescence compared with that in the water‐treated control at 5 dpi (Figure 7B). Consistently, immunoblot analysis revealed substantially reduced accumulation of the PVY‐GFP after JA treatment (Figure 7C). RT‐qPCR analysis yielded similar results, confirming the inhibitory effect of JA treatment on PVY accumulation (Figure 7D). Collectively, these results indicate that JA contributes to NbPSK3‐mediated resistance to PVY.

**FIGURE 7:**
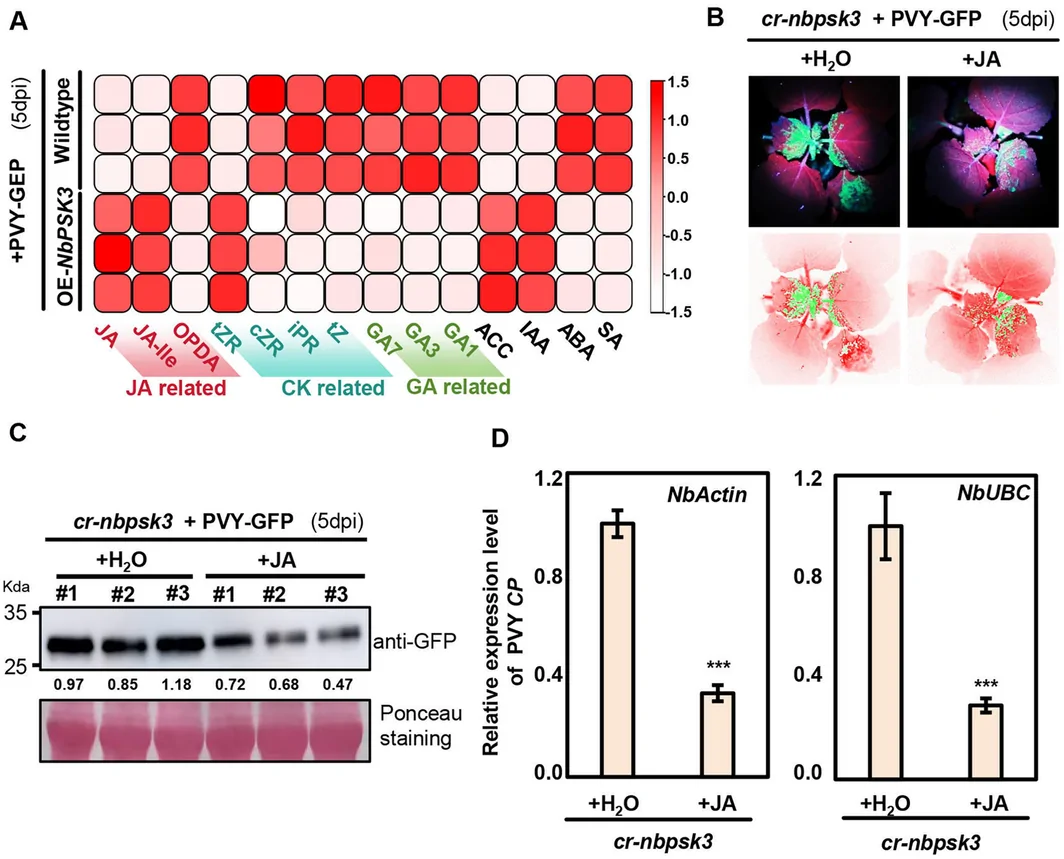
Jasmonic acid (JA) contributes to NbPSK3‐mediated antiviral resistance. (A) Heatmap showing phytohormone profiles in PVY‐GFP‐infected wild‐type (WT) and OE‐*NbPSK3* plants at 5 days post‐inoculation (dpi). Hormones and related metabolites were grouped into jasmonate (JA)‐, cytokinin (CK)‐, gibberellin (GA)‐, ethylene (ACC)‐, auxin (IAA)‐, abscisic acid (ABA)‐, and salicylic acid (SA)‐related classes. Three independent biological replicates were analysed for each genotype. (B) PVY‐GFP fluorescence in systemic leaves of *cr‐nbpsk3* plants treated with water or 10 mM JA at 5 dpi. (C) Immunoblot analysis of PVY‐GFP accumulation in water‐ and JA‐treated *cr‐nbpsk3* plants. (D) Reverse transcription‐quantitative PCR analysis of PVY *CP* accumulation in the corresponding plants. PVY *CP* accumulation was independently normalized to *NbActin* and *NbUBC*, shown in the left and right bar plots, respectively. “***” indicates significant difference (*p* < 0.01) in one‐way ANOVA. Each bar corresponds to the mean value ± SD of three biological replicates.

We tested the effects of OE‐*NbPSK3* on infection by TuMV and tobacco mosaic virus (TMV). Compared with the wild‐type, the OE‐*NbPSK3* plants presented markedly reduced TuMV infection (Figure 8A,B). Similar results were observed for TMV infection (Figure 8C,D). In addition, exogenous spraying assays were performed using synthetic NbPSK3‐DP peptide. Compared with water treatment, NbPSK3‐DP treatment substantially reduced infection by both viruses (Figure S9). These results indicate that NbPSK3‐DP has potential for broad‐spectrum resistance against multiple plant viruses.

**FIGURE 8:**
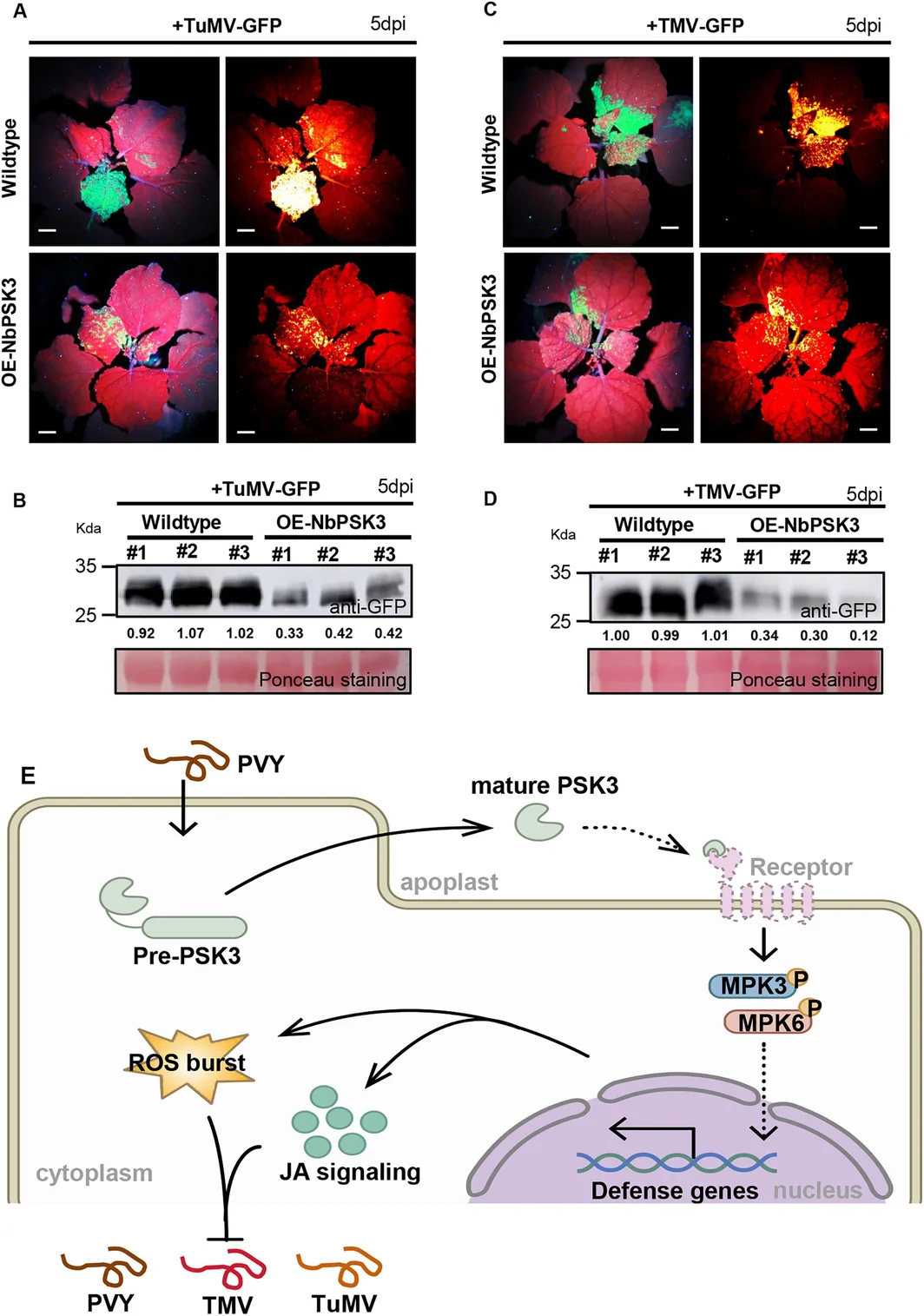
Resistance to TuMV and TMV and a proposed model of NbPSK3‐associated antiviral defence. (A) TuMV‐GFP fluorescence in systemic leaves of wild type and OE*‐NbPSK3* at 5 days post‐inoculation (dpi) under TuMV infection. Bar, 1 cm. (B) Western blot analysis of TuMV accumulation in the systemic leaves of wild type and OE*‐NbPSK3* using GFP antibody at 5 dpi. (C) TMV‐GFP fluorescence in systemic leaves of wild type and OE*‐NbPSK3* at 5 dpi under TMV infection. Bar, 1 cm. In (A) and (C), each lane represents a sample from an individual experiment. (D) Western blot analysis of TMV accumulation in the systemic leaves of wild type and OE*‐NbPSK3* using GFP antibody at 5 dpi. In (B) and (D), Ponceau S staining shows protein loading. Values beneath the bands indicate relative band intensities normalized to the corresponding loading control. Three samples are shown for each group. TuMV, turnip mosaic virus; TMV, tobacco mosaic virus. (E) Proposed working model for NbPSK3‐associated antiviral defence. NbPSK3‐DP, a non‐canonical peptide derived from the NbPSK3 precursor, activates MPK3/MPK6‐associated signalling and promotes reactive oxygen species (ROS) production, while NbSnRK2, NbACS1, and jasmonic acid (JA)‐associated responses also contribute to enhanced antiviral immunity.

## 3 Discussion

Plant viral diseases pose a major threat to global agricultural production, causing substantial yield losses (Jones and Naidu 2019; Scholthof et al. 2011). Owing to the obligate parasitic nature and rapid evolution of plant viruses, existing control strategies often fail to provide durable and broad‐spectrum protection, underscoring the urgent need to develop novel antiviral approaches based on a deeper understanding of host defence mechanisms (Quenouille et al. 2013; Tatineni and Hein 2023). The novel peptide hormone PSK is known for its diverse roles in regulating plant growth, development, and immune responses across multiple species (Li et al. 2024; Zhang et al. 2024). This study revealed that cultivar‐dependent resistance to PVY is accompanied by a distinct variation in the tobacco peptidome and identified NbPSK3‐derived noncanonical peptides as functional antiviral signals. By integrating comparative peptidomics across resistant and susceptible cultivars with peptide application assays and genetic perturbation of a PSK precursor gene, we established a causal link between a noncanonical PSK peptide hormone and multiple tested antiviral immune mechanisms. The resulting model extends the emerging concept of phytocytokines beyond bacterial and fungal pathosystems into plant–virus interactions.

Peptidogenomics has already been shown to be valuable for identifying peptides at the genome scale in microorganisms and humans (Kersten et al. 2011; Liu et al. 2011; Nguyen et al. 2013; Slavoff et al. 2013; Mohimani and Pevzner 2016; Mohimani et al. 2018). Wang, Tian, et al. (2020) established an integrated peptidogenomic pipeline and identified almost 2000 nonconventional peptides in both maize and *Arabidopsis*. The majority of these peptides originated from nonannotated regions, including intergenic sequences, introns, and untranslated regions (UTRs), rather than from canonical precursor genes (Wang, Tian, et al. 2020). Recently, Ali et al. (2024) constructed a comprehensive atlas of 6100 nonredundant endogenous peptides across 13 maize tissues, further demonstrating that peptide expression patterns are largely decoupled from those of their source proteins, implying that distinct regulatory mechanisms operate at the peptide level. Moreover, peptidomics has demonstrated considerable potential in the discovery of plant immune peptides. A novel peptidomics strategy identified wound‐ and MeJA‐induced defence signalling peptides in tomato, including a PR‐1‐derived peptide that enhanced antipathogen defence and showed minor antiherbivore activity (Chen et al. 2014). In addition, using a transcriptomics‐ and proteomics‐based pipeline, Wang, Yao, et al. (2020) identified 236 rice SSPs induced by *M. oryzae* and chitin, including the novel IRP, which activates *PAL1* expression and MAPK signalling in rice immunity. More broadly, recent studies have emphasized that integrated peptidogenomic and MS‐based workflows are becoming central to plant peptide discovery while also underscoring persistent barriers arising from low peptide abundance, post‐translational modifications, and incomplete plant‐specific databases (Xiao et al. 2025; Zhang et al. 2025; Wang et al. 2026). However, reports on the use of peptidomics to identify plant‐associated immune peptides are lacking, particularly ones comparing different disease‐resistant varieties. In our study, 31,260 peptides were identified across the three tobacco accessions with contrasting levels of PVY resistance, with PCA clearly separating samples by resistance phenotype, indicating that PVY resistance in tobacco is accompanied by a distinct variation in the endogenous peptidome. Moreover, 2892 peptides were specifically shared by the two resistant accessions, and 3199 peptides showed at least 1.5‐fold greater accumulation in resistant plants. These findings indicate that comparative peptidomics provides an effective strategy for identifying resistance‐associated endogenous peptides and support the existence of a distinct peptide regulatory layer underlying antiviral immunity in tobacco.

Although comparative peptidomics revealed clear resistance‐associated peptide patterns in tobacco, the relatively small number of annotated signalling peptides recovered also highlights an important technical limitation in current peptide discovery workflows. In maize and *Arabidopsis*, the adoption of six‐frame translation databases rather than conventional annotated protein databases dramatically expanded the recoverable peptide space by enabling the identification of nonconventional peptides encoded in nonannotated regions; the same pipeline was subsequently applied to grape, where large‐scale NCP discovery revealed widespread occurrence in noncoding genomic loci with developmental specificity (Pei et al. 2022). The NCPbook database was further constructed to compile noncanonical peptides from multiple plant species and facilitate their functional annotation (Sami et al. 2024). It has been well established that a primary constraint in the recovery of canonical signalling peptides through peptidomics is the incompleteness of current reference databases, particularly in nonmodel plants where precursor gene annotations remain sparse (Wang, Tian, et al. 2020; Pei et al. 2022). Notably, only nine peptides derived from three prepropeptides were classified into known peptide families (including RALF, PSK, and GASA) in this study, likely owing in part to the incomplete annotation of peptide precursor genes in nonmodel crops, which limits the ability of standard mass spectrometry search engines to accurately assign authentic peptides to their corresponding precursors. The adoption of peptidogenomic strategies—combining six‐frame translation databases with high‐resolution LC–MS/MS, as demonstrated in maize and grape—would be expected to substantially increase the recovery of canonical and noncanonical signalling peptides in tobacco and may uncover additional antiviral effectors beyond those identified here.

In addition to database limitations, the low representation of mature secreted signalling peptides in whole‐leaf extracts reflects the fundamental biological properties of this class of molecules. Canonical secreted peptides such as PSK are present at subnanomolar concentrations in the apoplast and are rapidly turned over upon receptor binding (Matsubayashi and Sakagami 1996; Komori et al. 2009). Moreover, PSK precursor genes in rice, including *OsPSK4*, *OsPSK5*, and *OsPSKR2*, are expressed at the highest levels in leaves (Liu, Zhang, Zeng, et al. 2025). The spatial and developmental specificity of peptide expression further limits peptide detectability in bulk tissue extracts: Long‐distance signalling peptides such as CEP and CLE‐RS are detectable only in xylem sap peptidomics (Okamoto et al. 2016), and the maize peptide atlas demonstrated that no single peptide maintained continuous expression throughout the 13 surveyed tissues and developmental stages (Ali et al. 2024). Thus, whole‐leaf extraction at a single infection time point inevitably dilutes signals from specific cell types and apoplastic compartments, where secreted peptides are likely to accumulate. In future studies, the integration of extracellular fluid enrichment strategies and gentle extraction methods that minimize mechanical disruption (Luo et al. 2019), combined with multiple time‐point sampling following viral infection, is expected to markedly improve the recovery of authentic antiviral secreted‐peptides from crops and enhance the efficiency of their functional characterization.

PSK signalling exerts context‐dependent and bidirectional effects on plant immunity across different host–pathogen systems (Table S2). In bacterial interactions, PSK–PSKR signalling attenuates PTI and promotes susceptibility to *P. syringae* pv. *tomato* in *Arabidopsis*, whereas *pskr1* mutants display enhanced bacterial resistance (Igarashi et al. 2012; Mosher et al. 2013). In contrast, PSK signalling positively contributes to antibacterial resistance in rice, where OsPSKR1 and the OsPSK–OsPSKR2 module increase resistance to bacterial leaf streak and bacterial blight, respectively (Yang et al. 2019; Liu, Zhang, Zeng, et al. 2025). In fungal pathosystems, PSK signalling is generally associated with enhanced resistance, although its effects remain host dependent. In *Arabidopsis*, *pskr1* mutants are more susceptible to the necrotrophic fungus *A. brassicicola* (Mosher et al. 2013), whereas in tomato, PSK–PSKR1 signalling promotes Ca^2+^‐dependent auxin responses and enhances resistance to *B. cinerea*, with PUB13‐mediated PSKR1 ubiquitination negatively fine‐tuning this response (Zhang et al. 2018; Hu et al. 2023). Positive effects have also been reported in rice resistance to blast disease and in cotton resistance to *Verticillium dahliae* (Zhang et al. 2022; Liu, Zhang, Zeng, et al. 2025). Notably, however, *V. dahliae* can also exploit PSK signalling in *Arabidopsis* and *N. benthamiana* through the effector VdSCP8, which promotes PSKR1–BAK1 signalling and suppresses PTI, further illustrating the context dependence of this pathway (Hua et al. 2025). In viral interactions, canonical sulphated PSKα has been shown to increase resistance to TuMV in *N. benthamiana* in a NbPSKR1‐dependent manner (Xiang et al. 2025). Together, these studies demonstrate that the immune output of PSK signalling is determined not only by pathogen type but also by the specific host–pathogen combination and downstream signalling network involved. A particularly important finding of this study is that NbPSK3‐DP does not correspond to the canonical sulphated PSKα pentapeptide YIYTQ but instead originates from a distinct region of the NbPSK3 precursor. This observation suggests that the biological information encoded by PSK precursors may extend beyond the canonical PSK motif. This scenario has precedents in other plant peptide systems. A single hydroxyproline‐rich systemin precursor can generate multiple biologically active defence peptides, whereas GmSubPep represents a cryptic defence peptide embedded within a relatively large subtilisin‐like protein (Pearce et al. 2001, 2010). Similarly, quantitative peptidomics revealed the immune‐regulatory peptide CAPE1 within the C‐terminal region of tomato PR‐1, and regions of Prosystemin outside the canonical Systemin sequence have been shown to retain biological activity (Chen et al. 2014; Molisso et al. 2022). Our identification of NbPSK3‐DP therefore raises the possibility that PSK precursors similarly harbour additional bioactive regions. However, whether NbPSK3‐DP is generated through a specific proteolytic maturation process and whether it is perceived by canonical PSK receptors remain to be determined.

The growth–immunity trade‐off remains a major challenge in improving crop disease resistance, as enhanced immune capacity often comes at the expense of plant growth and yield because of competition for shared energy resources and signalling pathways (Eichmann and Schäfer 2015; Ogawa‐Ohnishi et al. 2022). Notably, the overexpression of *OsPSK4/5* or *OsPSKR2* increased resistance to both rice blast and bacterial blight without causing significant changes in plant height, effective tiller number, grain length, grain width, or thousand‐grain weight compared with those of the wild type, suggesting that PSK signalling components can, under some conditions, strengthen immunity without obvious growth penalties in rice (Liu, Zhang, Zeng, et al. 2025). In tomato, PSK signalling has also been shown to modulate defence through the phosphorylation of the glutamine synthetase GS2 at specific serine residues, thereby linking PSK‐mediated immunity with nitrogen metabolism and growth regulation in a phosphorylation site‐dependent manner (Ding et al. 2023). In our study, *cr‐nbpsk3* mutants exhibited a bushier phenotype with significantly increased lateral branching, whereas *NbPSK3* overexpression did not significantly affect plant height, branch number, or seed weight. Moreover, the resistance of the OE‐*NbPSK3* plants to PVY, TuMV, and TMV was increased. Taken together, these findings suggest that NbPSK3 contributes to the regulation of shoot branching under normal growth conditions, whereas its overexpression is associated with increased resistance to multiple tested plant viruses without obvious developmental or yield‐related penalties under the conditions tested. Therefore, NbPSK3 may be a promising candidate for improving antiviral resistance in crops, although its effects on agronomic performance will require further evaluation under diverse genetic backgrounds and field conditions.

PSK signalling has been reported to activate multiple immune outputs, including ROS production, MAPK cascades, PR gene expression, and hormone crosstalk, across diverse plant–pathogen systems (Zhang et al. 2018; Liu, Zhang, Wang, and Wang 2025; Xiang et al. 2025). For example, *OsPSKR2* overexpression increased ROS‐ and PR‐related immune responses and blast resistance without obvious agronomic penalties in rice, whereas PSK–PSKR signalling promoted ROS and Ca^2+^ signalling to delay TuMV infection in a NbPSKR1‐dependent manner in *N. benthamiana* (Liu, Zhang, Liu, et al. 2025; Xiang et al. 2025). In addition, flg22‐triggered activation of PSKR1 and relief from BAK1‐mediated suppression placed PSK signalling downstream of bacterial PAMP perception, further supporting its role in amplifying PTI responses in *Arabidopsis* (Alqarni et al. 2026). Consistent with these observations, our results indicate that NbPSK3‐associated antiviral defence involves MAPK, ROS, and hormone‐related signalling. Exogenous NbPSK3‐DP rapidly activated MAPK phosphorylation and induced a transient ROS burst within minutes, whereas NbPSK3‐DP‐induced ROS accumulation was not detectable in *mpk3* or *mpk3^−/−^mpk6‐*RNAi plants, supporting a requirement for MPK3/MPK6‐mediated signalling in this response. Transcriptome analysis further revealed the enrichment of defence‐, MAPK‐, and hormone‐related pathways, and *NbSnRK2* and *NbACS1* were found to be functionally involved in NbPSK3‐mediated resistance. In addition, phytohormone profiling revealed coordinated changes in JA‐related metabolites, and exogenous JA treatment significantly reduced PVY accumulation in *cr‐nbpsk3* plants, indicating that JA‐associated signalling also contributes to NbPSK3‐mediated antiviral defence. Collectively, our results support a model in which the NbPSK3 precursor‐derived peptide activates MPK3/MPK6‐dependent signalling and ROS production, while also engaging in JA‐associated defence responses to enhance antiviral immunity (Figure 6E).

Broad‐spectrum antiviral resistance is a highly desirable yet difficult trait to engineer in crops because most known resistance mechanisms depend on the recognition of specific viral proteins or RNA sequences (Mandadi and Scholthof 2013). In this context, PSK‐mediated immunity may offer an alternative mode of antiviral defence. Notably, the activation of PSK–PSKR signalling by betulinic acid delayed infection not only by TuMV but also by TMV and cotton leaf curl Multan virus (CLCuMuV) in *N. benthamiana*, suggesting that this pathway may function against viruses from different families with distinct replication strategies (Xiang et al. 2025). Consistent with these observations, in our study, *NbPSK3* overexpression markedly reduced the accumulation of TuMV and TMV in addition to that of PVY, while the exogenous application of PSK3 also suppressed TMV and TuMV infection. Together, these findings suggest that NbPSK3‐mediated antiviral defence is unlikely to rely on the sequence‐specific recognition of individual viral components but is more likely associated with the enhancement of conserved host immune outputs. This feature may increase its potential to provide broader resistance than classical R gene‐mediated strategies, although the downstream signalling components involved and the identity of the PSKR(s) responsible for these antiviral effects remain to be clarified. More broadly, plant‐derived peptides, as endogenous regulators of growth, development, and stress adaptation, are increasingly regarded as safer and more environmentally friendly alternatives to conventional chemical pesticides for crop protection (Keerthana et al. 2025; Liu, Zhang, Wang, and Wang 2025; Yu et al. 2025). Recent perspectives have further emphasized their value as candidate biopesticides and biostimulants while also highlighting translational bottlenecks associated with peptide stability, delivery efficiency, large‐scale biomanufacturing, and regulatory evaluation (Yu et al. 2025; Wang et al. 2026). Our results indicate that both the genetic enhancement of NbPSK3 and the exogenous application of NbPSK3‐DP can suppress viral infection in *Nicotiana* without obvious agronomic penalties under the conditions tested, highlighting the potential practical value of NbPSK3‐based strategies for antiviral crop protection. Nevertheless, several limitations still need to be addressed before such strategies can be translated into field application. The stability, delivery efficiency, and formulation of exogenously applied peptides require further optimization. Finally, the durability of NbPSK3‐mediated resistance against rapidly evolving PVY strain complexes, as well as its effectiveness under mixed infection conditions involving other pathogens, remains to be determined.

## 4 Experimental Procedures

### 4.1 Plant Materials and Virus Sources

Three *N. tabacum* cultivars with contrasting responses to PVY (*Potyvirus yituberosi*), namely, the resistant cultivars CS01 and Coker176 and the susceptible cultivar NC95, were used for comparative peptidomic analysis. For the initial resistance screening of tobacco germplasm, a field isolate of PVY collected from naturally infected tobacco plants in Hunan Province (hereafter PVY‐HN), China, was used. This isolate was maintained in the Plant Pathology Laboratory of Hunan Agricultural University. For subsequent functional assays, the GFP‐tagged PVY infectious clone pCamPVY‐GZ‐GFP, derived from the PVY‐GZ isolate, was kindly provided by Prof. Xiangdong Li (Shandong Agricultural University, China) (Cheng et al. 2020). The parental PVY‐GZ isolate was classified as belonging to the SYR‐I strain in the original publication (Cheng et al. 2020). For functional assays, *N. benthamiana* was used as a model system because of its high amenability to transient expression and molecular validation. Both *mpk3* ^*−/−*^ and *mpk3* ^*−/−*^ *mpk6‐RNAi* were constructed as described by Feng et al. (2024). The TuMV (*Potyvirus rapae*) and TMV (*Tobamovirus tabaci*) used in the antiviral assays were maintained in our laboratory (Liu, Zhang, Liu, et al. 2025).

### 4.2 Generation of Transgenic Plants

OE‐*NbPSK3* plants were constructed using constitutive expression of the *NbPSK3* gene with a GFP tag (pRI101‐GFP) in *N. benthamiana*. Western blots with anti‐GFP antibody (1:5000; TransGen Biotech) were performed to screen the positive OE*‐NbPSK3* transgenic plants. Additionally, we generated *cr‐nbpsk3* lines by using CRISPR/Cas9 technology to knockout *NbPSK3* in *N. benthamiana*. Sanger sequencing revealed sequence variation at the *NbPSK3* target site in *cr‐nbpsk3* plants.

### 4.3 Growth Conditions and Virus Inoculation

Tobacco and *N. benthamiana* seeds were sown in moist nursery substrate and grown in a controlled growth chamber at 25°C ± 2°C under a 16 h light/8 h dark photoperiod. For comparative peptidomics, uniformly growing tobacco seedlings at the 5‐ to 6‐leaf stage were mechanically inoculated with PVY‐HN. Symptomatic systemic leaves collected from PVY‐HN‐infected source plants were homogenized in 20 mM phosphate buffer (pH 7.2), filtered through four layers of gauze, and used immediately as inoculum. Recipient leaves were lightly dusted with 600‐mesh quartz sand, gently rubbed with the inoculum along the veins, rinsed with ultrapure water, and returned to the growth chamber. For functional assays in *N. benthamiana*, pCamPVY‐GZ‐GFP was delivered by *Agrobacterium tumefaciens* ‐mediated infiltration. Local inoculated and systemically infected leaves were collected at the indicated time points (generally 5 and 8 dpi, respectively) for fluorescence imaging, RNA expression, and protein analyses. TuMV and TMV were inoculated using the laboratory‐maintained virus materials described above, and the sampling times are specified for each assay in the corresponding figure legends.

### 4.4 Peptide Extraction and Purification

Systemic leaves from PVY‐inoculated tobacco plants were collected at 8 dpi. For each cultivar, three independent biological replicates were prepared, with each replicate consisting of pooled leaves from three plants. The leaf samples were immediately frozen in liquid nitrogen and ground into a fine powder. For peptide extraction, 150 mg of powder was mixed with 200 μL of precooled 1% trifluoroacetic acid (TFA) and vortexed thoroughly. The homogenate was filtered through gauze and Miracloth and then centrifuged at 10,000 *g* for 20 min at 4°C. The supernatant was adjusted to a pH of 4.5 with 10 M NaOH to precipitate contaminants and then centrifuged again under the same conditions, after which the clarified supernatant was readjusted to a pH of 2.5 with TFA. The extract was concentrated under vacuum and freeze‐dried.

The dried material was dissolved in 1 mL of 0.1% TFA with ultrasonic assistance. After centrifugation, the supernatant was transferred to a precooled 10‐kDa ultrafiltration device to remove high‐molecular‐weight proteins. The filtrate containing the peptides was further desalted using MonoSpin C18 columns. Bound peptides were washed with 0.1% TFA and eluted with 50% acetonitrile containing 0.1% TFA. Eluates were vacuum‐dried and divided into two aliquots: one for peptide quantification using a peptide assay kit based on absorbance at 214 and 280 nm, and the other for LC–MS/MS analysis after resuspension in formic acid to a final concentration of 0.5 μg/μL. The samples were subsequently centrifuged and stored at −80°C until analysis.

### 4.5 LC–MS/MS‐Based Peptidomic Profiling

Purified peptide samples were subjected to LC–MS/MS analysis on a Q Exactive HF–X mass spectrometer coupled to an ultrahigh‐performance liquid chromatography system. Data acquisition was performed by Shanghai Applied Protein Technology Co. Ltd. Raw spectra were searched in PEAKS Studio v. 10.6 against the *N. tabacum* reference proteome obtained from UniProt (https://www.uniprot.org/). The search parameters were as follows: precursor ion mass tolerance, 10 ppm; fragment ion mass tolerance, 0.02 Da; maximum missed cleavages, 2; no enzyme specificity; and variable modifications including methionine oxidation, protein N‐terminal acetylation, and tyrosine sulfation, with up to two variable modifications allowed per peptide. Peptide identifications were filtered using a target‐decoy strategy at a false discovery rate (FDR) < 1%. The identified peptides were assigned to precursor proteins and categorized into known signalling peptide families or peptides derived from proteins with unknown functions.

### 4.6 Peptidomic Data Analysis and Candidate Peptide Screening

Peptide abundance matrices were generated on the basis of MS peak areas. Reproducibility among biological replicates was evaluated using Pearson correlation coefficients and visualized with the R package “pheatmap”. Principal component analysis (PCA) was performed with FactoMineR to assess global differences among cultivars. Peptides detected in all biological replicates of a group were retained for occurrence‐pattern analyses unless otherwise indicated.

Venn diagrams were generated using the VennDiagram package to visualize shared and cultivar‐specific peptides. For resistance‐associated comparisons, Coker176 and CS01 were considered the resistant group, and NC95 was considered the susceptible group. Peptides were classified as R‐specific or S‐specific according to their occurrence pattern and as R‐enriched or S‐enriched when their abundance differed by at least 1.5‐fold between the resistant and susceptible groups. Differential abundance was assessed using *t*‐tests with Benjamini–Hochberg correction, and the resulting fold changes and FDR‐adjusted significance values were visualized using volcano plots. To focus on candidate secreted peptide precursors, the precursor proteins were analysed with SignalP v. 6.0 using the plant Sec/SPI model, and proteins ≤ 300 amino acids containing a predicted N‐terminal signal peptide were retained. Functional enrichment analyses were performed on the corresponding precursor proteins, and candidates derived from known signalling‐peptide families were prioritized for biological validation.

### 4.7 Peptide Synthesis and Exogenous Peptide Treatment

Candidate peptides were chemically synthesized by Sangon Biotech (Shanghai, China) at ≥ 98% purity and desalted before use. The synthetic NbPSK3 precursor‐derived peptide (NbPSK3‐DP; initially designated PSK #2) matched the sequence and modification state assigned by LC–MS/MS and is distinct from the canonical PSKα YIYTQ motif. Lyophilized peptides were dissolved in sterile ultrapure water to prepare stock solutions and diluted to the indicated working concentrations immediately before treatment. Two‐week‐old *N. benthamiana* plants were infiltrated from the abaxial side with a needleless 1 mL syringe, and pCamPVY‐GZ‐GFP inoculation was performed 6 h after peptide treatment unless otherwise stated. Water‐treated plants served as controls.

### 4.8 DAB Staining

For histochemical detection of sustained ROS accumulation, leaves treated with NbPSK3‐DP or water were sampled at 4, 6, and 8 h after treatment. These time points were selected to monitor ROS accumulation after the rapid early ROS burst was measured by chemiluminescence. The samples were incubated in freshly prepared DAB solution (1 mg/mL, pH 3.8), vacuum infiltrated, and incubated in the dark. Chlorophyll was removed with 95% ethanol at 80°C, and H_2_O_2_‐dependent brown precipitates were documented under identical imaging conditions. To assess MAPK dependence, wild‐type, *mpk3*, and *mpk3^−/−^mpk6‐*RNAi leaves were treated with pCamPVY‐GZ‐GFP and processed using the same DAB staining procedure.

### 4.9 Chemiluminescence‐Based Detection of ROS

ROS production was measured using a chemiluminescence assay according to a previously described method with modifications (Schwacke and Hager 1992). Leaf discs were collected from 4‐ to 6‐week‐old plants and incubated overnight in sterile distilled water. Before detection, the water was replaced with a reaction mixture containing 10 mM L‐012 (120‐04891; WAKO), 0.5 mM horseradish peroxidase (HRP; Jackson Immunoresearch), and NbPSK3‐DP. flg22 (100 nM) and water served as positive and negative controls, respectively. Chemiluminescence was immediately measured using a GloMax 20/20 luminometer and recorded every 30 s for 90 min. Three biological replicates were performed for each treatment.

### 4.10 Protein Extraction and Immunoblot Analysis

Leaf tissues were ground in liquid nitrogen and homogenized in protein extraction buffer at a ratio of 100 μL of buffer per 0.1 g of tissue. The extracts were incubated on ice for 30 min with intermittent mixing and clarified by centrifugation at 14,000 *g* for 5 min at 4°C. The supernatants were mixed with 5× SDS loading buffer, denatured at 100°C for 10 min, separated by SDS–PAGE, and transferred to nitrocellulose membranes. The membranes were stained with Ponceau S to verify comparable loading, blocked with 3%–5% skim milk in phosphate‐buffered saline (PBS), and incubated with the indicated primary antibodies followed by HRP‐conjugated secondary antibodies. The signals were developed by enhanced chemiluminescence. PVY‐GFP accumulation was detected with an anti‐GFP antibody, and band intensities were quantified with ImageJ and normalized to the corresponding loading control. Immunoblot experiments were performed with three independent biological replicates.

### 4.11 Sequence Analysis

The precursor sequence corresponding to the candidate peptide PSK#2 was retrieved from the NCBI database (GenBank: Niben101Scf05193g01006.1). The candidate peptide was mapped to the precursor sequence and subsequently designated NbPSK3‐DP because it originates from an NbPSK3‐like precursor. Open reading frames were examined with NCBI ORF Finder, and N‐terminal signal peptides were predicted with SignalP v. 6.0. PSK precursor sequences from *Arabidopsis* and *N. benthamiana* were aligned for phylogenetic analysis, which was performed in MEGA using the neighbour‐joining method with 1000 bootstrap replicates.

### 4.12 RNA Extraction, cDNA Synthesis, and PCR Amplification

Total RNA was isolated from 50 to 100 mg of frozen *N. benthamiana* leaf tissue using the HiPure Plant RNA Mini Kit (Magen). The tissue powder was homogenized in RLC buffer, clarified by centrifugation, passed through a genomic‐DNA removal column, mixed with ethanol, and loaded onto a silica membrane. The column was sequentially washed with the kit wash buffers, and the RNA was eluted with RNase‐free water and stored at −80°C. RNA concentration and purity were assessed spectrophotometrically, and RNA integrity was checked before reverse transcription.

First‐strand cDNA was synthesized using the HiScript III 1st Strand cDNA Synthesis Kit (Vazyme). RNA was first denatured, treated with gDNA wiper mix, and reverse transcribed in a 20 μL reaction according to the manufacturer's protocol. The coding sequence of the PSK#2 precursor gene was amplified by RT‐PCR using gene‐specific primers. PCR products were analysed by agarose gel electrophoresis and used for subsequent cloning. The primers used are listed in Table S3.

### 4.13 Vector Construction and Transient Expression

The PCR products were purified with a HiPure Gel DNA Recovery Kit (Magen). For fluorescence‐based localization, full‐length NbPSK3 and the signal‐peptide‐deleted variant (NbPSK3^ΔSP^) were cloned and inserted into the indicated mCherry expression vector and introduced into *A. tumefaciens* GV3101. AtPIP1 was used as an apoplastic marker, and a plasma membrane marker NbCERK was coexpressed where indicated. Infiltrated *N. benthamiana* leaves were examined 48–72 h after agroinfiltration using a Leica TCS SP8 confocal microscope. Plasmolysis was induced before imaging to distinguish plasma membrane‐associated fluorescence from fluorescence retained in the apoplastic space. In parallel, secretion was biochemically verified using FLAG‐tagged NbPSK3 constructs and apoplastic fluid isolation, as described below.

### 4.14 Apoplastic Fluid Isolation and Secretion Validation

Apoplastic fluid was isolated from *N. benthamiana* leaves transiently expressing the inserted genes using a centrifugation‐based procedure adapted from Liu, Zhang, Wang, and Wang (2025); Liu, Zhang, Zeng, et al. (2025); Liu, Zhang, Liu, et al. (2025). Leaves were gently positioned inside a 50 mL syringe barrel, which was placed with the outlet facing downwards into a 50 mL centrifuge tube, and centrifuged at 2000 *g* for 20 min at 4°C. The recovered apoplastic fluid was immediately placed on ice for analysis. FLAG‐tagged full‐length NbPSK3, NbPSK3^ΔSP^, and the corresponding controls were analysed in both apoplastic and total‐protein fractions by immunoblotting with an anti‐FLAG antibody. A cytosolic marker was included to assess contamination of the apoplastic fraction.

### 4.15 Transcriptome Sequencing and Differential Expression Analysis

For transcriptome analysis, leaves from wild‐type and OE‐*NbPSK3* *N. benthamiana* plants were harvested at 5 dpi after pCamPVY‐GZ‐GFP inoculation. Total RNA was extracted as described above and subjected to quality assessment by agarose gel electrophoresis and spectrophotometry. RNA library construction and Illumina high‐throughput sequencing were performed by LC‐Bio Technologies (Hangzhou) Co. Ltd. Raw reads were filtered to remove adaptor sequences and low‐quality reads, generating clean data for downstream analysis. Clean reads were aligned to the *N. benthamiana* reference genome using HISAT2. DEGs were identified with DESeq2, using *q* < 0.05 and |log_2_FC| ≥ 1 as significance thresholds. Functional annotation of the DEGs was performed using UniProt, followed by Gene Ontology (GO) and Kyoto Encyclopaedia of Genes and Genomes (KEGG) enrichment analyses using the R package clusterProfiler.

### 4.16 MAPK Activation Assay

The MAPK activation assay was performed as previously described (Gao et al. 2022). Leaves were treated with NbPSK3‐DP or the indicated controls and collected at the specified time points. Total proteins were extracted in buffer containing 50 mM Tris–HCl (pH 7.5), 150 mM NaCl, 0.5% Triton X‐100, 1× protease inhibitor cocktail, 1 mM Na₃VO₄, 1 mM NaF, and 20 mM β‐glycerophosphate. Equal amounts of protein were resolved by SDS‐PAGE and immunoblotted with anti‐phospho‐p44/42 MAPK (pTEpY) antibody (1:2000; Cell Signalling Technology) to detect activated MPK3/MPK6. Ponceau S staining and/or the indicated total‐protein control were used to verify comparable loading.

### 4.17 qPCR

For qPCR, cDNA was diluted 5–10‐fold and amplified using ChamQ SYBR qPCR Master Mix (Low ROX Premixed; Vazyme) on a QuantStudio 5 real‐time PCR system. Gene‐specific primers, including primers targeting the PVY *coat protein* (*CP*) gene, are listed in Table S3. Reactions were performed with three biological replicates and technical replicates for each sample. The relative transcript abundance was calculated using the 2^−ΔΔ*C*t^ method. For PVY quantification, expression was normalized against that of two validated *N. benthamiana* reference genes (*Actin* and *UBC*) to minimize normalization bias.

### 4.18 TRV‐Based Gene Silencing of *NbSnRK2* and *NbACS1*

For the functional validation of *NbSnRK2* and *NbACS1*, gene‐specific fragments were subsequently cloned and inserted into the pTRV2 vector and introduced into *A. tumefaciens* GV3101. Cultures carrying pTRV1 and the corresponding pTRV2 construct were mixed and infiltrated into OE‐*NbPSK3* plants. The silencing efficiency was verified by RT‐qPCR before the PVY‐GFP challenge. The plants with confirmed silencing were inoculated with PVY‐GFP, and viral accumulation was evaluated at 5 dpi by GFP fluorescence imaging, immunoblotting, and RT‐qPCR of the PVY *CP*.

### 4.19 Phytohormone Profiling and JA Treatment

Phytohormone profiles were compared between PVY‐GFP‐infected wild‐type and OE‐*NbPSK3* plants. Systemic leaves were collected at 5 dpi, with three independent biological replicates for each genotype, and subjected to targeted LC–MS/MS analysis by Shanghai Applied Protein Technology Co. Ltd. (Shanghai, China). The hormone classes analysed included jasmonates, cytokinins, gibberellins, ABA, SA, auxin‐related metabolites, and 1‐aminocyclopropane‐1‐carboxylic acid (ACC).

To validate the function of JA‐associated signalling, *cr‐nbpsk3* plants were sprayed with 10 mM JA prepared in sterile water. JA was applied twice during the week before PVY‐GFP inoculation and once again after virus inoculation. Control plants were treated with sterile water. Viral accumulation was assessed at 5 dpi by GFP fluorescence imaging, immunoblotting, and RT‐qPCR.

### 4.20 Statistical Analysis

Significance analyses were performed by using SPSS Statistics 17.0 software (IBM Corp.). Significant differences among samples were determined using one‐way ANOVA. The Pearson correlation coefficient was estimated using the R function ‘cor. test’.

## Author Contributions

**Bin Yong:** methodology, software, formal analysis, visualization, writing – review and editing, writing – original draft, project administration, validation, conceptualization, funding acquisition. **Shiming Lin:** methodology, data curation, formal analysis, writing – original draft, validation. **Xiaowei Liu:** validation, software, formal analysis. **Peng Liu:** formal analysis. **Tianye Zhang:** validation. **Kaili Zhong:** methodology. **Jiaqian Liu:** methodology. **Qianjun Tang:** resources, visualization, methodology. **Xiangping Zhou:** methodology, data curation. **Hailin Cai:** methodology, data curation. **Tianbo Liu:** supervision, funding acquisition, writing – review and editing, project administration, conceptualization, validation. **Jian Yang:** writing – review and editing, funding acquisition, supervision, project administration, conceptualization, validation, methodology.

## Funding

This work was supported by the grants from the Hunan Science and Technology Project (HN2024KJ03), the National Natural Science Foundation of China (32502508).

## Conflicts of Interest

The authors declare no conflicts of interest.

## Supporting information


**Figure S1:** Disease resistance index of PVY infection in different tobacco varieties.

**Figure S2:** The length and molecular weight of the small peptides detected by peptidomics.

**Figure S3:** Classification of resistance‐ vs. susceptibility‐ associated peptide precursor proteins.

**Figure S4:** Reverse transcription‐quantitative PCR analysis of PVY accumulation following candidate‐peptide treatment.

**Figure S5:** Identification of *NbPSK3*‐edited plants by Sanger sequencing.

**Figure S6:** Reverse transcription‐quantitative PCR analysis of PVY accumulation in *NbPSK3*‐overexpressing and knockout plants.

**Figure S7:** Transcriptome analysis of *NbPSK3*‐overexpressing plants under PVY infection.

**Figure S8:** Validation of *NbSnRK2* and *NbACS1* silencing in OE‐NbPSK3 plants.

**Figure S9:** Exogenous application of NbPSK3 could reduce the infection of tobacco by various viruses.

**Table S1:** Gene ontology (GO) terms of differentially expressed genes.

**Table S2:** Context‐dependent roles of PSK signalilng in plant immunity.

**Table S3:** Primers used in this work.

## Data Availability

All data are available in the manuscript and the supplementary materials are provided in Supporting Information online. The RNA‐seq data have been uploaded at the NCBI SRA database (https://www.ncbi.nlm.nih.gov/sra) under the BioProject accession no. PRJNA1459595.
