# Supplementary material for: Comparative Peptidomic Identification of a Noncanonical PSK3 Precursor‐Derived Peptide That Enhances Antiviral Defence Through MAPK‐Dependent ROS Signalling in Tobacco
Source: Mol Plant Pathol. 2026 Nov 11;27(11):e70361. doi: 10.1111/mpp.70361 (PMC13755522; doi:10.1111/mpp.70361)
Supplement: Supplementary file 1 [file MPP-27-e70361-s001.pdf]

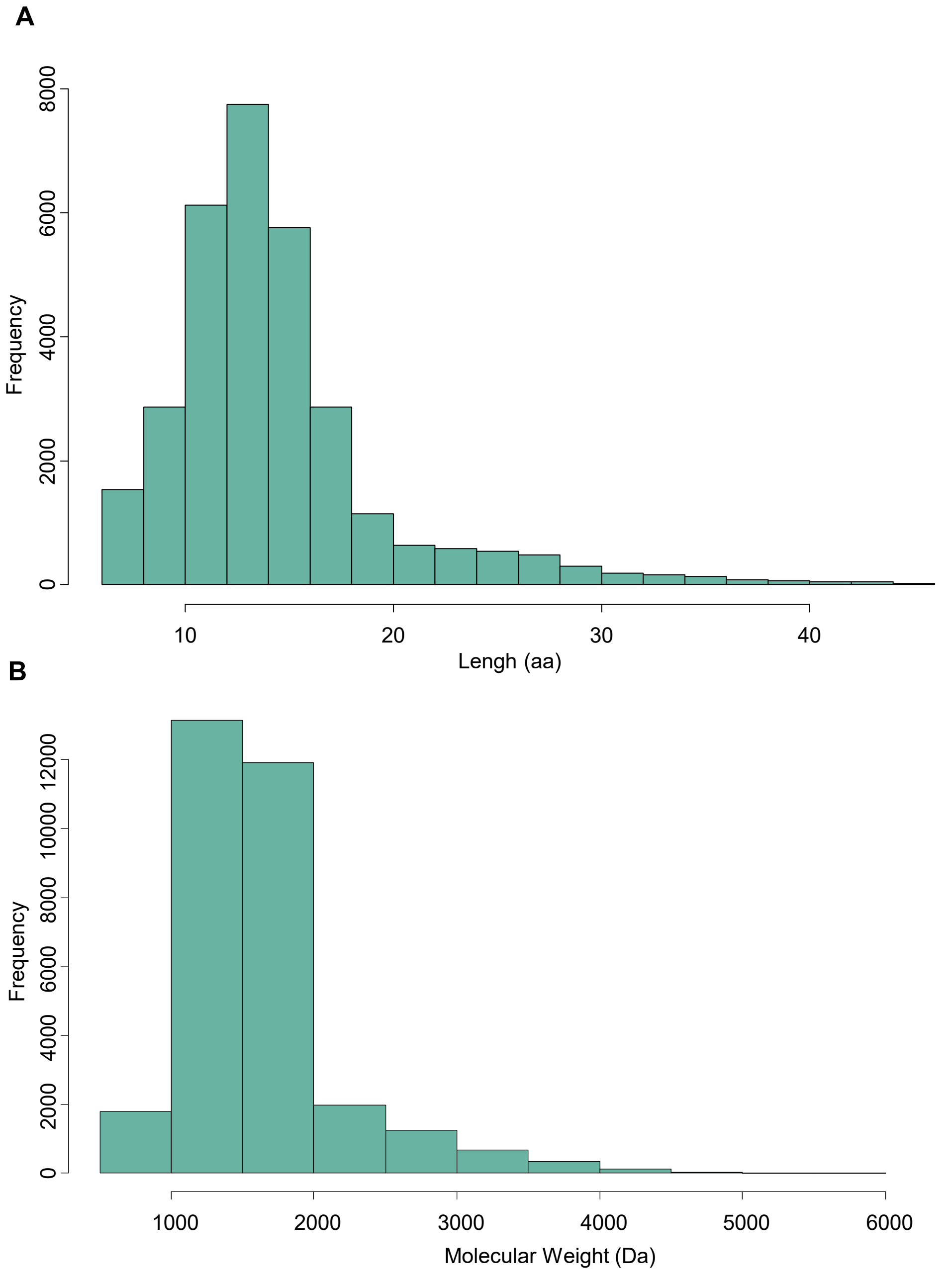

Figure S2 The length and molecular weight of the small peptides detected by peptidomics. (A) Frequency distribution of peptide lengths, expressed as numbers of amino acid residues. (B) Frequency distribution of peptide molecular masses, expressed in daltons (Da). The distributions summarize the peptides identified in the comparative peptidomic analysis of PVY-infected tobacco cultivars.
