# Supplementary material for: Comparative Peptidomic Identification of a Noncanonical PSK3 Precursor‐Derived Peptide That Enhances Antiviral Defence Through MAPK‐Dependent ROS Signalling in Tobacco
Source: Mol Plant Pathol. 2026 Nov 11;27(11):e70361. doi: 10.1111/mpp.70361 (PMC13755522; doi:10.1111/mpp.70361)
Supplement: Supplementary file 2 [file MPP-27-e70361-s003.pdf]

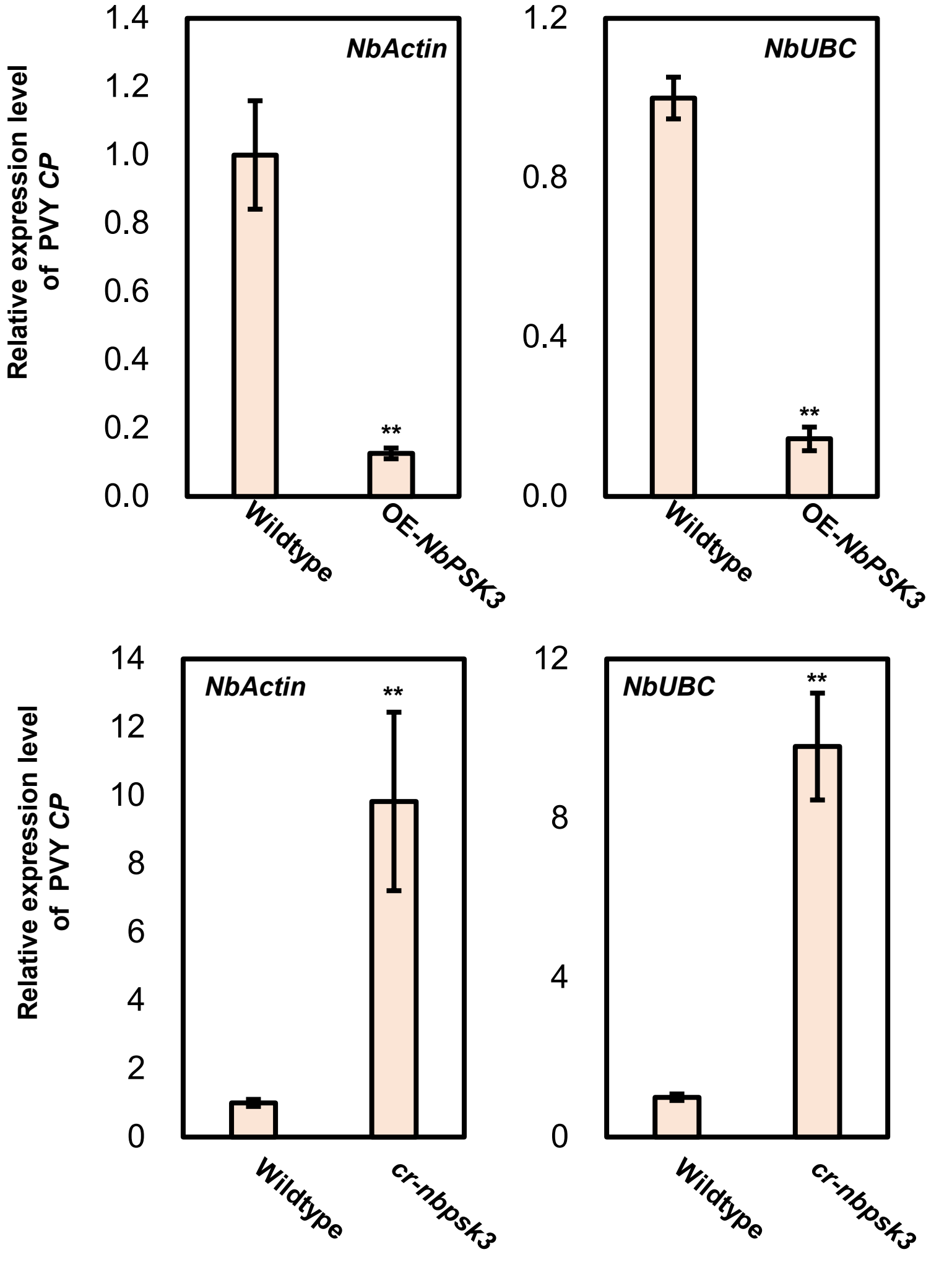

Figure S6. qRT-PCR analysis of PVY accumulation in NbPSK3-overexpressing and knockout plants. Relative Potato virus Y *coat protein* (PVY *CP*) RNA abundance in systemic leaves of wild-type (WT), NbPSK3-overexpressing (OE-*NbPSK3*) and NbPSK3 knockout (*cr-nbpsk3*) *Nicotiana benthamiana* plants at 5 days post-inoculation (dpi) with PVY-GFP. The upper pair of plots compares WT and OE-*NbPSK3* plants, and the lower pair compares WT and *cr-nbpsk3* plants. PVY *CP* RNA abundance was measured by RT-qPCR and normalized independently to *NbActin* (left) and *NbUBC* (right), with the corresponding WT control set to 1. Bars represent means ± SD of three biological replicates. Differences from the corresponding WT control were assessed using t-tests; $^{**}P < 0.01$.
