# Supplementary material for: Comparative Peptidomic Identification of a Noncanonical PSK3 Precursor‐Derived Peptide That Enhances Antiviral Defence Through MAPK‐Dependent ROS Signalling in Tobacco
Source: Mol Plant Pathol. 2026 Nov 11;27(11):e70361. doi: 10.1111/mpp.70361 (PMC13755522; doi:10.1111/mpp.70361)
Supplement: Supplementary file 3 [file MPP-27-e70361-s004.pdf]

Y48	9.65	R	ZY2010	48.55	MS	HC2030	61.11	S
▶ Coker176	12.58	R	CZ-59	49.02	MS	CZ-67	62.22	S
VAM	19.82	R	K326	49.44	MS	YZ09	62.78	S
CS03	20.89	MR	CS07	50	MS	YZ2008	63.89	S
CS19	21.48	MR	CZ-67	50.39	MS	HNCS04	63.89	S
CZ66	21.6	MR	ZY1803	52.64	MS	21HC20	64.58	S
G140	25.61	MR	HC2032	53.33	MS	HC2232	69.44	S
CS05	25.89	MR	C81-3- 5	53.33	MS	HNCS19	69.89	S
CS06	32.54	MR	ZY2021	53.7	MS	XND2302	70.83	S
G28	33.57	MR	CS08	55	MS	CZ62	72.22	S
YZ-02	34.44	MR	云烟87	55	MS	HC2033	72.92	S
Ti145	37.4	MR	YZ10	55	MS	CZ79	72.92	S
▶ CS01	38.63	MR	E48	55.07	MS	YZ09	73.61	S
ZY2011	40.35	MS	HC2233	55.56	MS	HC2108	74.31	S
ZY1702	42.15	MS	CS12	57.22	MS	20619	74.31	S
CS19	43.61	MS	ZY1804	58.21	MS	2008	75	S
XN2002	45.45	MS	CZ81-2	60.07	S	XL01	75	S
ZY2023	45.87	MS	HC2119	60.22	S	XND2301	75.63	S
C81-2-2	47.1	MS	M87	60.56	S	CZ83	76.39	S
20122	47.12	MS	HC2111	60.93	S	▶ NC95	82	S

Figure S1 Disease resistance index of PVY infection in different tobacco varieties. Disease indices and resistance classifications of 60 Nicotiana tabacum accessions following infection with the PVY-HN field isolate. Lower disease indices indicate lower disease severity. Red triangles mark Coker176, CS01 and NC95, the cultivars selected for comparative peptidomic analysis. R, resistant; MR, moderately resistant; MS, moderately susceptible; S, susceptible.
