# Supplementary material for: Comparative Peptidomic Identification of a Noncanonical PSK3 Precursor‐Derived Peptide That Enhances Antiviral Defence Through MAPK‐Dependent ROS Signalling in Tobacco
Source: Mol Plant Pathol. 2026 Nov 11;27(11):e70361. doi: 10.1111/mpp.70361 (PMC13755522; doi:10.1111/mpp.70361)
Supplement: Supplementary file 4 [file MPP-27-e70361-s005.pdf]

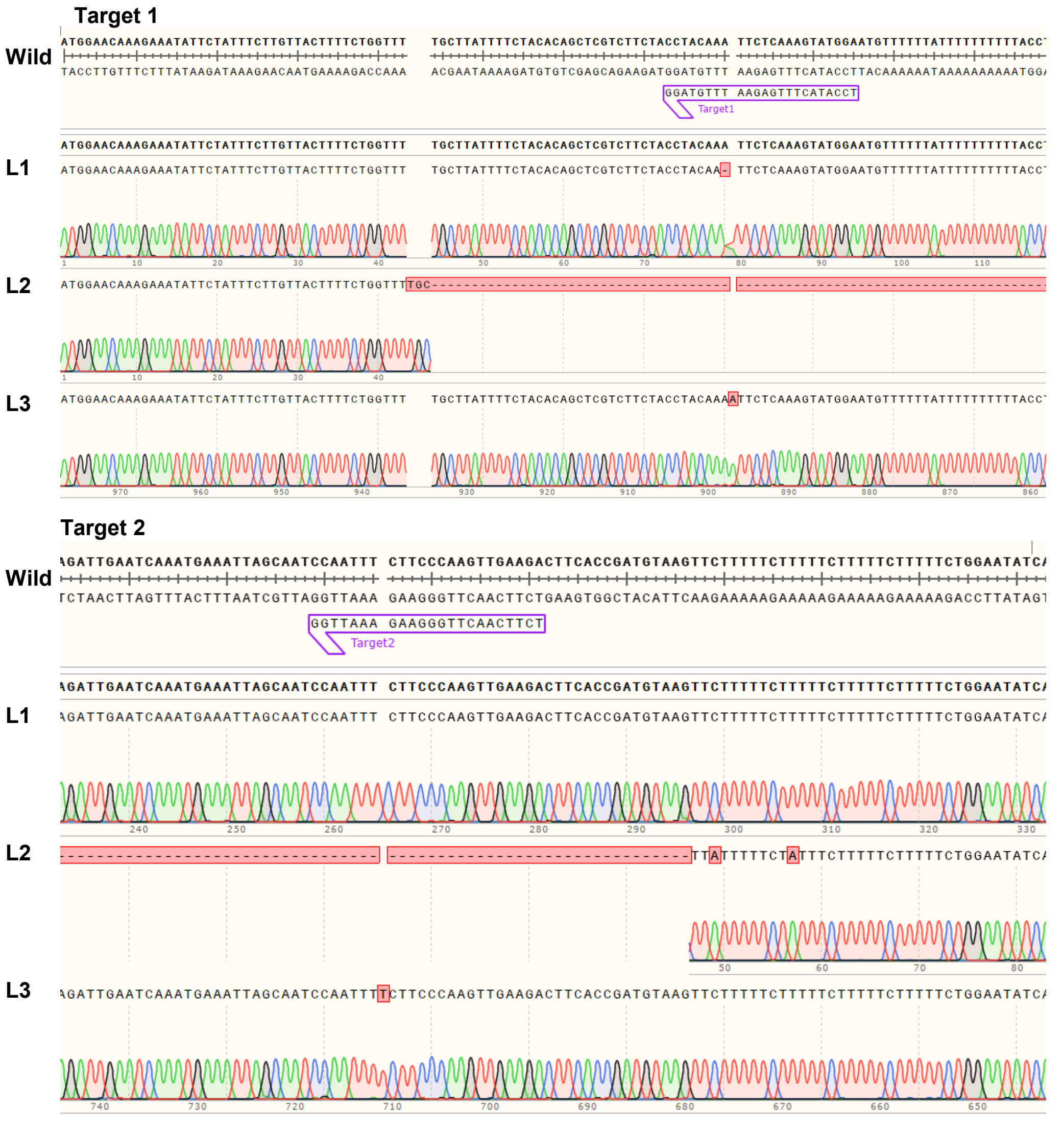

Figure S5 Identification of *NbPSK3*-edited plants by Sanger sequencing. Sanger sequencing traces and sequence alignments of wild-type (WT) and three NbPSK3-edited plants (L1–L3) at the two target regions. Target 1 and Target 2 are shown in the upper and lower panels, respectively. The target regions are annotated, and sequence differences relative to WT are highlighted. These data document sequence changes in the edited plants used to characterize the cr-nbpsk3 genotype.
