# Supplementary material for: Comparative Peptidomic Identification of a Noncanonical PSK3 Precursor‐Derived Peptide That Enhances Antiviral Defence Through MAPK‐Dependent ROS Signalling in Tobacco
Source: Mol Plant Pathol. 2026 Nov 11;27(11):e70361. doi: 10.1111/mpp.70361 (PMC13755522; doi:10.1111/mpp.70361)
Supplement: Supplementary file 5 [file MPP-27-e70361-s006.pdf]

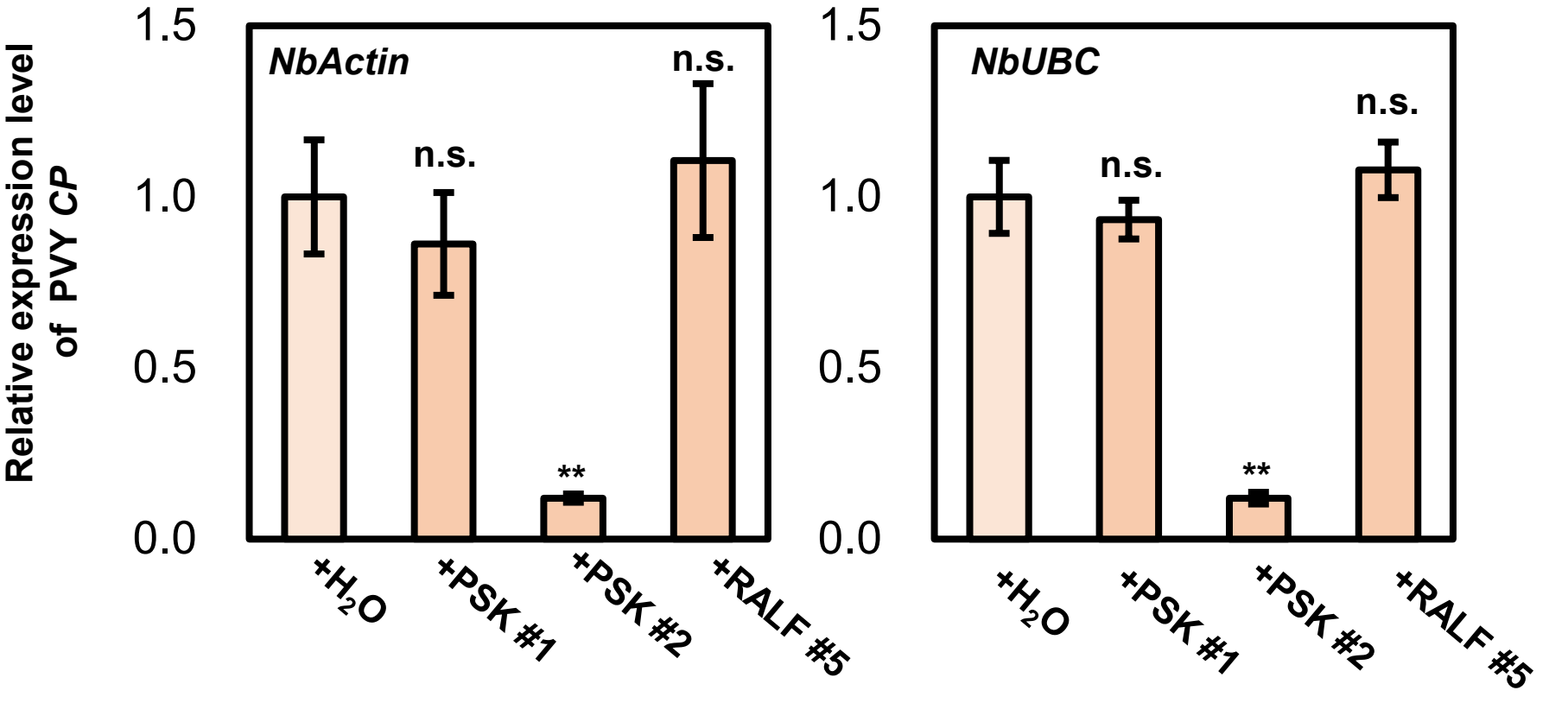

Figure S4. qRT-PCR analysis of PVY accumulation following candidate-peptide treatment. Relative Potato virus Y *coat protein* (PVY *CP*) RNA abundance in *Nicotiana benthamiana* leaves treated with PSK #1, PSK #2 or RALF #5 and challenged with PVY-GFP, measured at 5 days post-inoculation (dpi). H_2O served as the control. PSK #2 denotes the noncanonical NbPSK3-derived peptide, NbPSK3-DP. PVY CP RNA abundance was measured by reverse-transcription quantitative PCR (RT-qPCR) and normalized independently to *NbActin* (left) and *NbUBC* (right). Values are expressed relative to the H_2O control. Bars represent means ± SD of three biological replicates. Differences from the H_2O control were assessed using t-tests; $^{**}P < 0.01$; n.s., not significant.
