# Supplementary material for: Comparative Peptidomic Identification of a Noncanonical PSK3 Precursor‐Derived Peptide That Enhances Antiviral Defence Through MAPK‐Dependent ROS Signalling in Tobacco
Source: Mol Plant Pathol. 2026 Nov 11;27(11):e70361. doi: 10.1111/mpp.70361 (PMC13755522; doi:10.1111/mpp.70361)
Supplement: Supplementary file 6 [file MPP-27-e70361-s007.pdf]

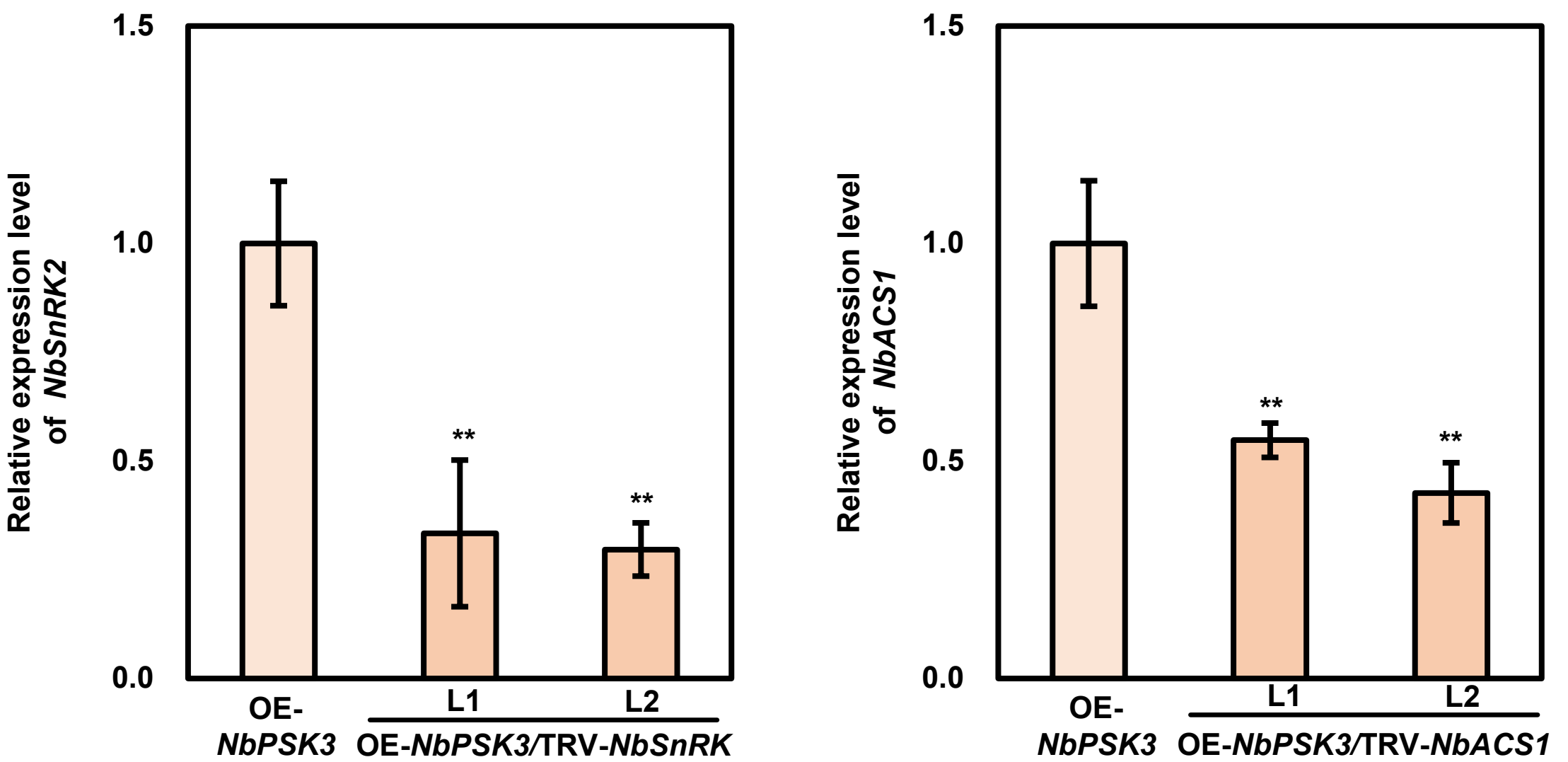

Figure S8. Validation of *NbSnRK2* and *NbACS1* silencing in OE-NbPSK3 plants. Relative *NbSnRK2* (left) and *NbACS1* (right) transcript abundance in *NbPSK3*-overexpressing (OE-*NbPSK3*) *Nicotiana benthamiana* plants following TRV-mediated silencing of the corresponding gene, measured by qRT-PCR. L1 and L2 denote two independently silenced plants for each target gene and correspond to those examined in Figure 5. Expression is shown relative to the corresponding OE-*NbPSK3* control, which was set to 1. Bars represent means ± SD (n = 3 biological replicates). Differences from the corresponding control were assessed using t-tests; $^{**}P < 0.01$.
