# Supplementary material for: Comparative Peptidomic Identification of a Noncanonical PSK3 Precursor‐Derived Peptide That Enhances Antiviral Defence Through MAPK‐Dependent ROS Signalling in Tobacco
Source: Mol Plant Pathol. 2026 Nov 11;27(11):e70361. doi: 10.1111/mpp.70361 (PMC13755522; doi:10.1111/mpp.70361)
Supplement: Supplementary file 7 [file MPP-27-e70361-s008.pdf]

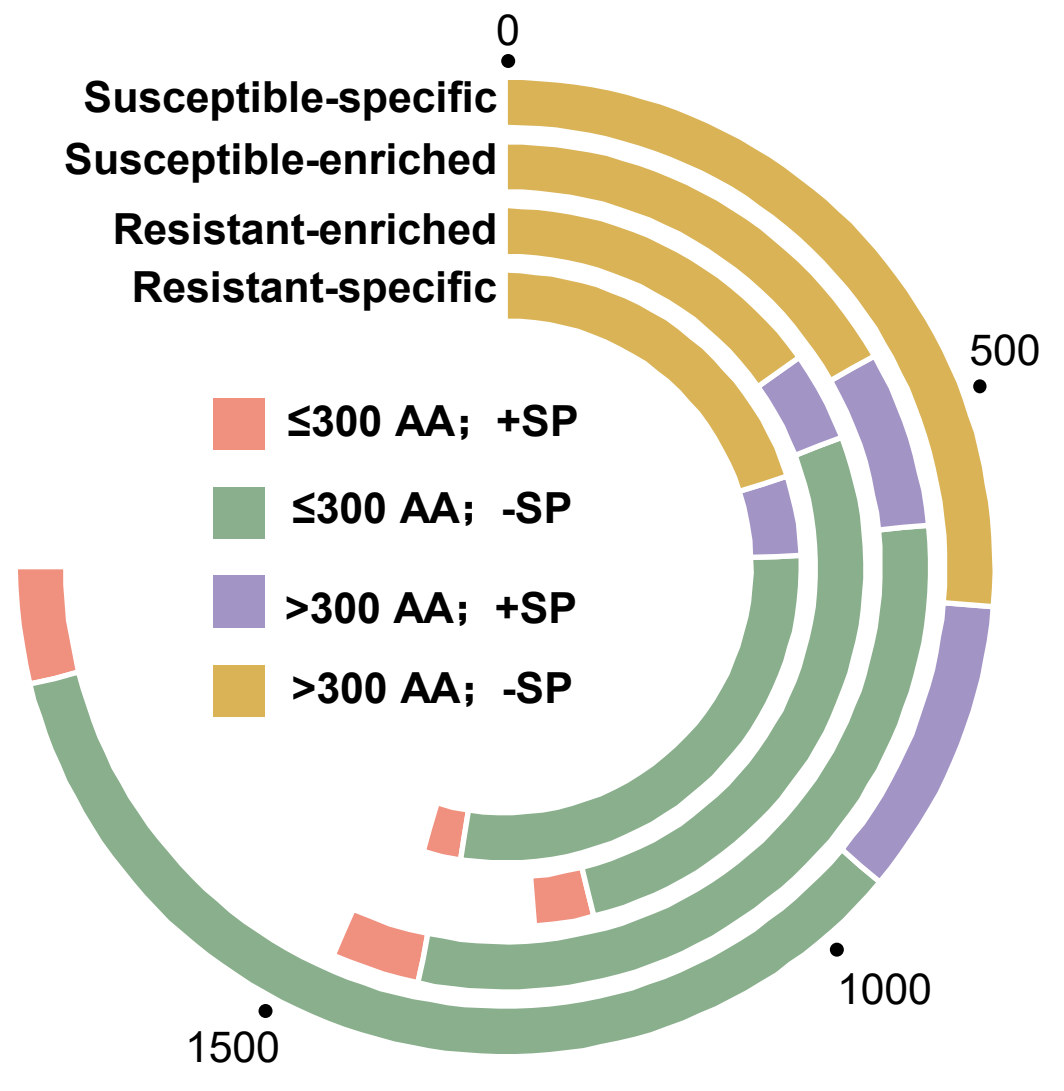

Figure S3. Classification of resistance-associated and susceptibility-associated peptide precursors. Precursor proteins corresponding to susceptible-specific, susceptible-enriched, resistant-enriched and resistant-specific peptides are shown from the outermost to the innermost ring, respectively. Within each group, precursors are classified by length (≤300 or >300 amino acids) and the presence (+SP) or absence (−SP) of a predicted signal peptide. Resistant-group peptides are associated with Coker176 and CS01, whereas susceptible-group peptides are associated with NC95. Specific peptides were detected only in the corresponding group, and enriched peptides showed at least 1.5-fold higher abundance in that group. SP, signal peptide.

。
