# Supplementary material for: Comparative Peptidomic Identification of a Noncanonical PSK3 Precursor‐Derived Peptide That Enhances Antiviral Defence Through MAPK‐Dependent ROS Signalling in Tobacco
Source: Mol Plant Pathol. 2026 Nov 11;27(11):e70361. doi: 10.1111/mpp.70361 (PMC13755522; doi:10.1111/mpp.70361)
Supplement: Supplementary file 8 [file MPP-27-e70361-s011.pdf]

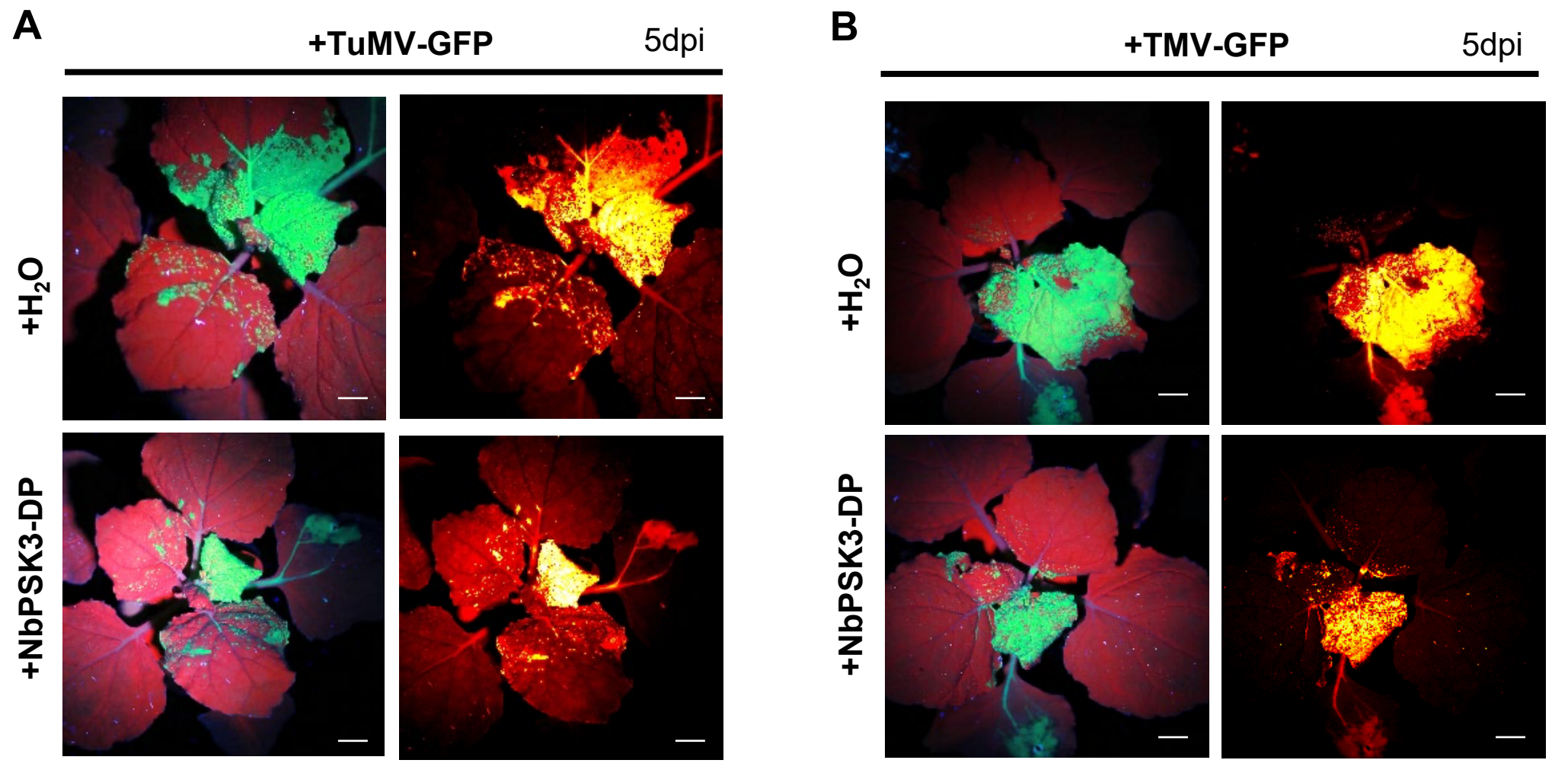

Figure S9 Exogenous application of NbPSK3 could reduce the infection of tobacco by various viruses. (A) Fluorescence analysis of TuMV infection 5 days post-infection (dpi) following exogenous application of NbPSK3-DP. (B) Fluorescence analysis of TMV infection 5 days post-infection (dpi) following exogenous application of NbPSK3-DP. TuMV, Turnip mosaic virus; TMV, Tobacco mosaic virus.
