# Supplementary material for: Comparative Peptidomic Identification of a Noncanonical PSK3 Precursor‐Derived Peptide That Enhances Antiviral Defence Through MAPK‐Dependent ROS Signalling in Tobacco
Source: Mol Plant Pathol. 2026 Nov 11;27(11):e70361. doi: 10.1111/mpp.70361 (PMC13755522; doi:10.1111/mpp.70361)
Supplement: Supplementary file 9 [file MPP-27-e70361-s012.pdf]

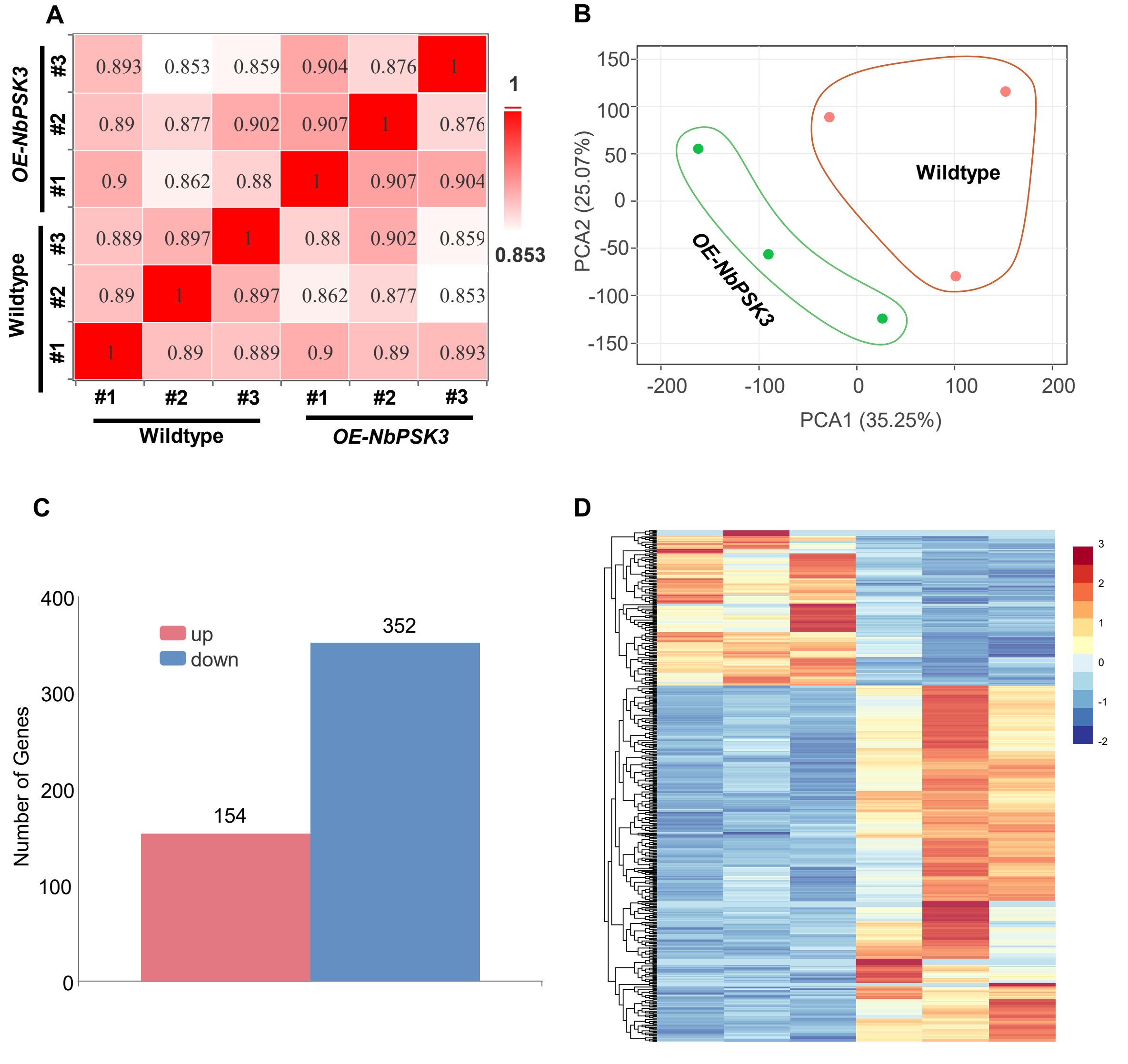

Figure S7 Transcriptome analysis of *NbPSK3*-overexpressing plants under PVY infection. Transcriptomes of wild-type (WT) and *NbPSK3-overexpressing* (*OE-NbPSK3*) *Nicotiana benthamiana* plants were compared at 5 days post-inoculation (dpi) with PVY-GFP, using three biological replicates per genotype. (A) Correlation between wild-type and *OE-NbPSK3* plant samples. (B) PCA analysis of plant samples from wild-type and *OE-NbPSK3* lines. (C) Identification of differentially expressed genes between wild-type and *OE-NbPSK3* plants. (D) Heatmap of differentially expressed genes between wild-type and *OE-NbPSK3* plants.
